# The perceived impact of early years interventions being delivered by volunteers: a qualitative evidence synthesis of volunteer experiences

**DOI:** 10.3389/fpubh.2026.1933122

**Published:** 2026-10-13

**Authors:** Louise Padgett, Sarah Louise Blower, Peter Murphy, Maria Bryant

**Affiliations:** 1 Department of Health Sciences, University of York, York, United Kingdom; 2 Centre for Health Economics, University of York, York, United Kingdom; 3 Department of Health Sciences and the Hull York Medical School, University of York, York, United Kingdom

**Keywords:** babies and infants, family support, infant, toddler (MeSH), volunteers

## Abstract

**Objective:**

To understand the views and experiences of volunteers who deliver early years interventions (EYIs) regarding their contributions to positive outcomes for families and the impact of volunteering on them personally.

**Methods:**

Qualitative and mixed-methods studies including the perspectives of volunteers within interventions supporting families in the first 1,001 days were identified through academic and gray literature searches. Direct quotes from volunteers and author interpretations were extracted, and the data analyzed using thematic synthesis methods.

**Results:**

A total of 28 studies covering the views of 387 volunteers delivering 32 different EYIs were included. Three analytical themes were identified: (1) Volunteers as a mechanism for change; (2) Supporting volunteers to succeed; and (3) The transformative power of volunteering for volunteers.

**Conclusions:**

Volunteers felt particularly well suited to delivering early years interventions by drawing on their personal experiences and building trust with families who may be hesitant to engage with statutory services. Volunteering also helped them develop professional and personal skills and enhanced their social wellbeing, suggesting benefits that extend beyond family outcomes to wider community values. However, adequate training and supervision are essential to ensure that volunteers feel confident in their roles and to minimize potential emotional harm.

## 1 Background

Effective early years interventions (EYIs), particularly those in the first 1,001 days (i.e., from conception to 2 years of age), are crucial in supporting the healthy development of children and preventing negative long-term impacts on their health and prospects (1–3). These interventions often involve supporting the mental wellbeing of parents, increasing parenting abilities, and supporting mothers with breastfeeding and attachment with their infant (4–8). Volunteers are a key part of many EYIs (9, 10). Reported reasons for adopting a volunteer model include volunteers being deemed more relatable, empathetic and likely to reach the target population of the intervention (11–13) while being a mode of increasing the reach and sustainability of the service through reducing costs (14–16). This is particularly important in the early years context, where interventions are typically delivered by voluntary or nonprofit organizations, often commissioned by statutory organizations with limited budgets for prevention and early intervention efforts (17, 18).

Evidence of the effectiveness of volunteer-led EYIs from randomized controlled trials of breastfeeding interventions, interventions to support the mental wellbeing of parents and parenting interventions (19–26) is mixed. Evidence from qualitative studies and service evaluations is more positive (11, 27–31), with studies reporting that volunteer-led EYIs are well received by parents and generate positive parenting and child development outcomes (32). However, it is less clear whether and why, volunteers feel suited to providing support, and how they believe they can contribute to positive outcomes for parents and infants.

Although not commonly reported, volunteers are also considered potential beneficiaries of volunteer-led EYIs (11, 33). It is viewed that volunteers can and should benefit from their volunteering roles (34), and that their inclusion in services can be the means of expanding the value of a service. Volunteering can provide pathways into employment through the development of skills (35), can improve mental wellbeing (36), reduce isolation and increase life satisfaction (37). However, research on volunteers within health and social care services has found that volunteers suffer from stress and burnout (38, 39), and that volunteers working with individuals undergoing hardship become distressed by their experiences (40, 41). Existing evidence of the impact of volunteering is often based on older volunteers (aged over 50 years) (42), or on volunteers from a broad range of settings (e.g., volunteering in sport and environmental settings). Research of the impact on volunteers within specific settings has been recommended to account for different volunteer characteristics and to understand the implications of including volunteers across different contexts (42). A rapid review conducted in 2015 of evaluations of volunteer-led interventions supporting child development in the early years has evidenced some benefits to volunteers in this setting, including volunteers gaining skills and improving confidence and self-esteem (10). However, not all studies related to interventions for parents in the first 1,001 days are reported, and the quality these studies are not reported.

A scoping review identified a recent increase in qualitative research exploring volunteers' experiences in EYIs, but this evidence has not been synthesized (9). Synthesizing these experiences could improve understanding of how and why volunteers contribute to positive outcomes for parents and infants, informing the design, delivery, and commissioning of volunteer-led EYIs. It could also identify the support volunteers need to perform their roles effectively while highlighting the benefits and challenges of volunteering. This evidence may also demonstrate the wider social value of volunteer-led EYIs through benefits that extend to the volunteers, supporting funding and commissioning decisions, particularly in the context of limited public resources (17, 18). In addition, understanding volunteers' experiences could inform strategies to improve recruitment, retention, and sustainability (43, 44), and help determine which EYIs are appropriate for volunteer delivery rather than for professional or statutory provision (45, 46).

The objectives of this review were to (1) explore how volunteers view and experience their role across different EYIs, and perceptions of how their roles can support positive outcomes to parents and their infants, and (2) explore if and how volunteers were positively and negatively impacted by their role. The experiences and views of volunteers involved in different interventions to support parents in the first 1,001 days (breastfeeding, mental wellbeing, parenting, doula, attachment, and transition-to-parenthood interventions) were included to explore similarities and differences in their experiences and impact across different roles. This can provide more specific consideration for commissioners of EYIs who may be considering the evidence base for a range of EYIs.

## 2 Methods

This review was planned and conducted using thematic synthesis methods as recommended in Qualitative Evidence Synthesis (QES) guidance (43) and is reported according to Enhancing Transparency in Reporting the Synthesis of Qualitative Research (ENTREQ) reporting standards (44). The review was registered on PROSPERO prior to final searches being conducted (PROSPERO reference: CRD42022301443).

### 2.1 Search strategy

Search terms were based around the Population, Phenomena of Interest, Context (PICo) framework (45) and informed by previous relevant reviews (9, 10, 46, 47), with terms related to the population of interest (i.e., volunteers), the phenomena of interest (i.e., the experiences, perceptions of and benefits to volunteers) and the context (i.e., EYIs). Qualitative study design terms were also included in the search, and database-specific qualitative and mixed-method search filters were applied to increase the specificity of searches (48–50). See Supplementary File for an example of search strategy.

The databases, Medline, Embase, CINAHL, PsycINFO, Web of Science, and The Applied Social Sciences Index and Abstracts (ASSIA) were initially searched in February 2022 and updated in March 2025. Gray literature sources were also searched to provide a more complete view of the available evidence and to be inclusive of perspectives presented outside peer-reviewed journals (51, 52). Gray literature was sought through relevant organization websites including Early Intervention Foundation (EIF) (now Foundations), National Institute for Health and Care Excellence (NICE), Department of Health, Nesta, Barnardo's, Action for Children, Family Action, Home Start and NSPCC. References within eligible papers and relevant reviews were checked for further eligible papers.

### 2.2 Eligibility criteria

The eligibility criteria were based on the population, phenomena of interest and context of the review, the study design, and type of evidence source. The same eligibility criteria were applied to gray literature and peer reviewed literature.

Population: studies had to include data collected from volunteers with roles in EYIs, with specific, extractable data. Data collected from other individuals involved in the intervention, such as service users, were not included. Studies that referred to a role as something other than a volunteer (i.e., peer or mentor) were only included if it was confirmed that these roles were voluntary and unpaid (receipt of expenses was acceptable).

Phenomena of interest: studies which explored the experiences, motivations or perceptions of volunteers who had taken on a role within an intervention to support parents of infants within the first 1,001 days (conception to 2 years) were included. Studies exploring perceived personal impact or experience of taking on their role were also included. Studies that focus only on how volunteers perceive the intervention's impact on the service user were not included.

Context: eligible interventions were those involving volunteers to support parents during the first 1,001 days delivered in the UK or comparable high-income countries. Interventions included those supporting the transition to parenthood, parenting (skills, confidence, and satisfaction), parent wellbeing (emotional and mental), breastfeeding, and support for attachment and parent-child relationships. Interventions could also include paid staff roles; however, interventions where volunteer roles were not distinct from paid roles were not included.

Study design: the review included both qualitative and mixed-methods studies in which qualitative findings could be extracted separately. Qualitative data collection and analysis methods must have been described.

Evidence source: studies were required to be available in English and published since 2000. Studies not published in English were eligible for inclusion, provided a translated copy could be obtained from the author to ensure the meaning of the data were accurately translated. The decision to focus on publications since year 2000 was guided by a rapid review conducted in 2016 (10) that included studies from 1990 onwards which identified only a limited number of potentially relevant studies from 1990–2000 (*N* = 5). Reviews, conference abstracts, dissertations and protocol papers were not included.

### 2.3 Evidence selection

Search results were managed in the EndNote software (53). Duplicate records were removed and studies were exported to Covidence (54). Two reviewers (LP and PM) screened the first 10% of records at title and abstract and full-text screening stages. Reasons for exclusion were recorded during the full-text screening stage. Inter-rater reliability was calculated using the Kappa statistical test (55). A very strong score (i.e., 0.80 or above) was achieved at title and abstract screening stage (0.85). At the full-text screening stage, no score above 0.80 was achieved. After discussions on the sources of disagreement (e.g., lack of clarity in the definition of the volunteer role), a second round of screening of a further 10% of full texts was conducted by both reviewers, and a very strong inter-rater reliability score was achieved (0.85). LP screened remaining records.

### 2.4 Data extraction

A Google Form was created and used to extract data for each study, including evidence source (i.e., country of study), intervention purpose and design, characteristics of the volunteers and their roles, study's objectives, data collection and analysis methods. First-order findings (direct quotes) and second-order findings (author interpretations) from results and findings sections of included articles (43) were extracted.

### 2.5 Quality appraisal

The Critical Appraisal Skills Programme (CASP) Qualitative Studies Quality Appraisal tool was used to assess the quality of included studies (56). The validity of the study results, the appropriateness of recruitment, data collection and analysis methods, and the contribution of each study were assessed. Two initial studies were independently appraised for quality by two reviewers (LP and SB) to ensure that the first reviewer (LP) was identifying the relevant information to appraise studies appropriately. The CASP tool was not designed to provide an overall score for the quality of papers, and therefore no overall scores have been reported. Studies were not excluded from the analysis based on quality to avoid excluding specific perspectives or deviant cases.

### 2.6 Analysis and synthesis

Findings were synthesized and analyzed using the thematic synthesis method (43). A three-step process was followed, in which all extracted data were coded deductively (e.g., based on the objectives of the review) or inductively (i.e., to explore new insights within the data) line by line, followed by grouping similar codes to generate descriptive themes. Descriptive themes were then considered and interpreted in relation to the review objectives to produce analytical themes (43). Throughout the analysis process, contextual factors (e.g., intervention purpose and the delivery style of the volunteer role) within individual studies were considered to understand whether findings were consistent across all contexts or specific to certain roles, volunteer characteristics, or interventions. Coding was conducted by the lead author (LP), and debriefings with the second authors (MB and SB) were carried out during theme development.

## 3 Results

Initial database searches conducted in February 2022 yielded 15 articles eligible for inclusion. Three further documents identified through citation and website searches also met criteria for inclusion. The authors of eight articles were contacted for clarification on whether those delivering the intervention were volunteers, resulting in two articles being included in the review. Eight articles were identified and included following an updated search in March 2025 (all sourced from academic databases). The final review included data from 28 articles or documents, reporting research on 32 different interventions (see Table 1). Abstracts of all non-English full-text publications were available in English, and none were deemed eligible. Figure 1 shows the results of searches and reasons for exclusion.

**Table 1:** Study characteristics.

Study details	Intervention purpose and target population	Intervention delivery style and duration	Intervention setting and funding	Volunteer characteristics	Volunteer role	Volunteer training and supervision	Study aim	Study methods (Qualitative components of mixed methods studies only)	Sample size and sample characteristics (volunteer sample only)
Abbott (68) UK	*Name:* not specified *Purpose:* to provide birth support to women in prison *Target population:* pregnant women in prison	*Delivery style:* In person within the prison *(In person one-on-one support)* *Duration/frequency*: In labor and *ad hoc* afterwards	*Intervention setting:* voluntary sector *Funding:* not reported	*Title:* Birth supporters (No further background details provided) *(Non-peer volunteers)*	To support women during birth and after if needed *(Provider of emotional support)*	*Training amount:* not reported Debriefing and supervision provided	To examine the experiences of birth supporters attending imprisoned women who are being separated from their babies.	*Methodology:* exploratory study, qualitative design *Data collection:* In-depth interviews *Data analysis:* thematic analysis	Sample size: 12 interview sample characteristics not reported.
Biggs (74) Australia	*Name:* national Perinatal Anxiety & Depression Helpline *Purpose: to support m*ental wellbeing through peer support. *Target population:* Mothers only during the perinatal period	*Delivery style:* Telephone support (*Remote supporter)* *Duration/frequency:* Support provided as and when needed.	*Intervention setting:* Nationally provided service through a not-for-profit organization. *Funding details: Not reported*.	*Title:* Peer supporter (Someone with personal experience of perinatal mental illness or supporting someone with perinatal mental illness) *(Peer)*	To provide peer support and counseling *(Provider of emotional support)*	*Training amount*: 10 h plus observations. Supervised (amount not reported)	To explore the experiences of women providing volunteer peer support on the helpline.	*Methodology:* Not stated *Data collection:* open-ended questionnaires and focus groups *Data analysis:* analyzed thematically, interpreted using the Empathy Altruism and the Helper Therapy Principle	*Sample size:* 11 online survey and 8 focus group participants Online survey sample: *age:* 30–39 years, *n* = 5 above 40, *n* = 6 *Education: completed degree or higher. n = 8, completed a certificate, diploma or apprenticeship, n = 2, Not reported (NR), n = 1*. *Time in volunteer role:* Less than a year, *N* = 3 1–3 years, *N* = 6 More than 3 years, *N* = 2 Focus group sample: *Age: 30–39 years, n = 3 Above 40 rears, n = 5* *Education: completed degree or higher, n = 4; completed a certificate, diploma or apprenticeship, n = 4* *Time in volunteer role:* less than a year, *N* = 5 1–3 years, *N* = 3 More than 3 years, *N* = 0
Carter et al. (75) UK	*Name:* not reported *Purpose:* Provide peer support to women with postnatal depression to improve outcomes. *Target population:* women identified as having postnatal depression	*Delivery style:* home visiting *(in-person one-to-one support)* *Duration/frequency: 6 sessions over 6 weeks*	*Intervention setting:* large teaching hospital *Funding:* not reported	*Title:* Peer support workers Women who have recovered from postnatal depression *(Peer volunteers)*	Supporting mother into a personal recovery program, promoting hope, providing support based on common experiences. *(Provider of emotional support)*	*Training amount: one* standalone training day Weekly group or individual supervision	To explore the lived experiences of peer support workers (PSWs) during their intervention with mothers suffering from postnatal depression (PND).	*Methodology:* not reported *Data collection:* log books completed by peer support workers after each visit with mothers and after each supervision. *Data analysis:* constant comparative approach	*Sample size:* 8 *Ethnicity:* all white British
Carter et al. (76) UK	*Name: not reported* *Purpose: support women with antenatal depression* *Target population:* first-time mothers with antenatal depression with no previous experiences of mental health issues, miscarriage, or stillbirth recruited during 28–30 weeks of pregnancy.	*Delivery style: in-person one-to-one sessions are a venue suited to both the volunteer and woman. (In-person one-to-one supporter)* *Duration/frequency: 6 weeks of 1 h sessions*	*Intervention setting: not specified*; *however, the NHS oversaw the references and Disclosure and Barring Service (DBS) processes for the volunteers* *Funding:* Not reported	*Title:* Peer Support Workers Women who have recovered from antenatal depression *(Peer volunteers)*	Support women as a fellow ‘mother to mother'. To listen, guide and provide support in ways that would have helped them when they were in a similar situation. *(Provider of emotional support)*	*Training amount: two* days Supervision provided, amount not reported	To assess the acceptability, recruitment, feasibility and effectiveness of a peer support intervention for women with antenatal depression.	*Methodology:* mixed methods. Qualitative methodology not stated *Data collection:* logbooks recording and reflecting on meetings with participants and semi-structured interviews. *Data analysis:* thematic analysis	*Sample size: 4* sample characteristics not reported.
Coe and Barlow (61) UK	*Name:* the Perinatal Support Project *Purpose:* to support mental wellbeing; parent-child attachment; reduce isolation; improve confidence of parents and improve confidence of volunteers *Target population:* mothers with mild-to-moderate mental health issues during pregnancy and up to the infant being aged 1 year.	*Delivery style:* home visiting and group sessions *(in-person one-to-one support and group support)* *Duration/frequency:* not reported	*Intervention setting*: provided by Family Action within the Voluntary sector *Funding:* not reported	*Title:* Volunteer befriender *(non-peer volunteers)*	To increase understanding and knowledge of the infant's needs and to help to develop informal support networks. *(Educator)*	*Training amount:* 6 days Ongoing supervision	To measure outcomes for service participants and volunteer befrienders engaged in the Perinatal Support Project (PSP) and to explore stakeholder (project coordinators, volunteers, service users and referrers to the service) perceptions, and experiences of delivering and engaging with the PSP.	*Methodology:* mixed methods. Qualitative methodology not stated *Data collection:* semi-structured interviews *Data analysis:* through a process of coding and analysis, common themes were identified	*Sample size:* 14 *Age:* range, 20-60 years, half over 40s *Ethnicity:* majority described themselves as white British All but one had children. Most participants were either married or in a stable relationship. Half of the sample had experience of postnatal depression.
Curtis et al. (27) UK	*Title: Breastfriends Doncaster* *Purpose:* to support mothers with breastfeeding *Target population:* mothers from low-income groups during the breastfeeding period	*Delivery style:* one-to-one (out-of-home setting) *(In-person one-to-one supporter)* *Duration/frequency:* Not reported	*Intervention setting:* not specified *Funding:* Department of Health	*Title: Breastfriends volunteers (Young, working-class women who had personal experience of breastfeeding) (Peer volunteers)*	To promote breastfeeding within their own social networks and with women identified through health professionals *(Educator)*	*Training amount:*20 h Ongoing telephone support	To explore how peer supporters and health professionals negotiated the provision of support for breastfeeding mothers	*Methodology:* descriptive qualitative study *data collection:* Focus groups *data analysis:* Thematic analysis	*Sample: size:* 7 *Characteristics:* not reported
Downie et al. (11) Australia	*Name:* Community Mothers *Purpose:* Improve confidence of parents and increase the physical and social health and cognitive development outcomes of the child *Target population:* First-time parents up to the infant being aged 1 year	*Delivery style:* Home visiting *(In-person one-to-one support)* *Duration/frequency:* 1 year	*Intervention setting:* a collaboration between the health service, a university and community health unit *Funding: Not reported*	*Title:* Community mothers (Mothers from the local community) *(Non-peer volunteers)*	To support and serve as a gateway to local parenting resources in the community. Role model, increase confidence, and empower parents. *(Educator, provider of emotional support, connector to further services)*	*Training amount:* Amount not specified Monthly support meetings	To report the perceptions and experiences of the volunteer home visitors engaged in the Community Mothers Programme.	*Methodology:* Not stated *Data collection:* Semi-structured interviews *Data analysis: Content analysis*	*Sample size:* 12 *Age: Range* 22 to 57 years (mean age of 34) All English-speaking Participants were mothers of between 1 and 6 children *Time in volunteering role: Range, 5 months to 4 years (mean, 18 months)*
Granville and Sugarman (33) UK	*Name:* Parents 1st. Pregnancy pals and Birth Buddies *Purpose:* To provide friendly support, to stay healthy, to have a positive birth, and to give their babies the best possible start. *Target population:* Parents with social, health or educational disadvantage during pregnancy and up to the infant being aged 3 months	*Delivery style:* Home visiting *(In-person one-to-one support)* *Duration/frequency: Support until the infant is aged 3 months*	*Intervention setting: delivered by a social enterprise* *Funding:* NHS South Essex	*Title:* Volunteer peer supporter *(Peer volunteer)*	Actively listening to parents' needs and priorities through a non-judgemental and respectful approach *(Provider of emotional support)*	*Training amount:* Amount not specified Supervised (amount not specified)	The focus of the fieldwork was to better understand the relationship between the parent and the volunteer peer supporter, and how that relationship brought about change.	*Methodology:* Mixed methods. Qualitative methodology not stated *Data collection:* In-depth interviews and focus groups *Data analysis: Thematic approach*	*Sample size:* 6 interview participants, focus group participant number not reported *Characteristics:* Not reported
Grimes et al. (77) Australia	*Name:* not specified *Purpose:* to support mothers with breastfeeding and adjustment to parenthood *Target population:* first-time mothers only from the birth of their infant to the infant being aged 6 months	*Delivery style:* telephone support *(Remote supporter)* *Duration/frequency:* weekly for 12 weeks	*Intervention setting:* not specified *Funding:* included within research funding	*Title:* Volunteers/peers (who had breastfed for at least 6 months and were not considered professionals) *(Peer volunteers)*	Provide peer support, emotional, informational and appraisal support, building confidence, showing empathy and signposting to services *(Educator, provider of emotional support, provider of appraisal support and connector to further support*.	*Training amount:* 4 h ongoing supervision	To explore the experiences of volunteers providing peer support to increase understanding of their motivation for becoming peer supporters, and to describe their perceptions and experiences, to understand factors that may impact the duration of participation.	*Methodology:* qualitative descriptive design guided by the Functional theory of volunteerism *Data collection:* focus groups *Data analysis:* thematic analysis that combined inductive and deductive techniques	*Sample size:* 17 *Age (years):* 30–34, *N* = 4 35–39 *n* = 9 40+ *N* = 4 Mean years, (SD) 37.1 (4.2) *Country of birth:* Australia, *N* = 12 England, *N* = 1 South Korea, *N* = 1 USA, *N* = 1 New Zealand, *N* = 1 Argentina, *N* = 1 *English as first language: N* = 14 *English as first language: N* = 14 *Partnered: N* = 16 *Average number of children: N* = 2 *Average age of youngest child—*18 months (range, 4–48 months) *Current employment:* employed—full time: *N* = 2 Employed—part time: *N* = 6 Maternity leave: *N* = 4 Home duties: *N* = 5 *Usual occupation:* education/teaching, *N* = 24 Health professional, *N* = 3 Professional (other), *N* = 3 Administrative and/or management, *N* = 3 ‘Stay-at-home' mother, *N* = 2 Others, *N* = 2 *Income:* $350–649 per week ($18,200–33,799 per year): *n* = 1 $650–999 per week ($33,800–51,999 per year): *n* = 1 $1,400–1,999 per week ($72,800–103,999 per year): *n* = 7 More than $2,000 per week ($104,000 or more per year): *n* = 7 NR: *n* = 1 *Education:* completed a degree or higher *n* =13 completed diploma or certificate *n* = 3 completed secondary school to year 12 (or equivalent) *n* = 1.
Hopper and Skirton (30) UK	*Name:* not specified *Purpose:* to support mothers with breastfeeding *Target population:* mothers shortly after birth	*Delivery style:* one-to-one on maternity ward *(in-person one-to-one support)* *Duration/frequency:* not reported	*Intervention setting:* NHS hospital *Funding:* not reported	*Title:* Peer supporters (Mothers who had breastfed) *(Peer volunteers)*	Share experiential knowledge of breastfeeding and provide informational, emotional, and appraisal support *(Educator, provider of emotional support, provider of appraisal support)*	*Training amount:* 25 Supervision details not reported	To establish the sustainability of a volunteer peer support service for new breastfeeding mothers in a hospital setting from the perspectives of the volunteers and ward staff.	*Methodology:* “Cross-sectional” qualitative descriptive study *Data collection:* Semi-structured interviews *Data analysis:* Thematic analysis	*Sample size:* 6 *Characteristics:* not reported
Kane Low et al. (57) USA	*Name:* the Doulas care program *Purpose:* improve maternal and child health outcomes of at-risk population groups by meeting the emotional and physical health needs of low-income women and adolescents with at risk pregnancies *Target population:* mothers who have a low income and/or are socially or medically at risk during pregnancy and postpartum	*Delivery style:* home visiting, support in labor and telephone support *(In-person one-to-one support and remote supporter)* *Duration/frequency:* not reported	*Intervention setting:* Provided by a community-based charity *Funding:* W. K. Kellogg Foundation	*Title:* Doula *(non-peer volunteer)*	Teaching parenting skills, breastfeeding education, practical support (i.e., childcare, housework), transportation to prenatal visits, accompaniment to prenatal classes, prenatal home visits, answers to questions about pregnancy, childbirth, and parenting; education to promote healthy behaviors throughout pregnancy, support during labor, assistance with breastfeeding. *(educator, provider of practical support)*	*Training amount:* 3 days Supervision details not reported	To assess what education they would need to perform their role as community-based doulas.	*Methodology:* not stated *Data collection:* focus groups *Data analysis:* content analysis	*Sample size:* 14 *Age:* range 25 to 48 years old (average: 34 years old) *Number of children: (range 1–3)* *Marital status:* 12 married and 2 single *Education:* 8 with some college education, 6 with college degree *Profession:* 9 considered themselves stay-at-home mums *Income: < $20,000 n = 4*, *>$50,000 n = 4 NR n = 6*
Khaw et al. (69) Australia	*Intervention name:* birth for Humankind *Purpose:* to provide ‘doula support' for underserved communities *Target population:* mothers experiencing financial disadvantage or no birth support, mental health challenges, family violence or trauma, homelessness, or of young age.	*Delivery style:* in person support including in hospital setting. *(In person one to one support)* *Duration/frequency:* support provided throughout pregnancy, labor and up to 12 weeks postpartum	*Intervention setting:* community based *Funding:* not reported	*Title:* Doula (Individuals with professional experience in birth support and have attended at least 3 births. Professional backgrounds included private practice doulas, student doulas, bicultural doulas, midwifery students, midwives, and former midwives) *(Non-peer volunteer)*	To provide continuous emotional, physical, and social support. *(Provider of emotional support)*	*Training amount:* trained (amount not reported).	To explore the roles of community-based doulas in providing culturally responsive care to migrant women in Australian maternity settings, from the perspectives of doulas and maternity care providers.	*Methodology:* a social constructivist epistemology using a qualitative interpretive phenomenological perspective guided this study. *Data collection:* semi-structured interviews *Data analysis:* thematic analysis	*Sample size:* 10 volunteer characteristics not reported separately
Khaw et al. (71) Australia							To explore how community-based doulas and health care providers perceive and experience working with each other when supporting migrant and refugee women during labor and birth and to understand the facilitators and barriers to positive and collaborative doula-provider working relationships	*Methodology:* a social constructivist epistemology, using a qualitative, interpretive, phenomenological perspective guided this study. *Data collection: in-depth s*emi-structured interviews *Data analysis: combined inductive and deductive analysis including t*hematic analysis and framework analysis using the Capability, Opportunity and Motivation (COM-B) model of behavior change	
Kuliukas et al. (62) Australia	*Name:* father-focused breastfeeding antenatal classes (FFAB) *Purpose:* to support fathers to support mothers with breastfeeding through peer support *Target population:* fathers who were involved in the Parent Infant Feeding Initiative before birth of their child	*Delivery style:* group session *(Group supporter)* *Duration/frequency:* One-off 50-min session	*Intervention setting:* provided across six public hospitals *Funding:* not reported	*Title:* Peer supporter (Fathers of at least one child aged 3 years or younger who was breastfed for a minimum of 3 months) *(Peer volunteers)*	Deliver class, provide peer support, educate in keeping with the philosophy and attributes of peer support (i.e., not trained as paraprofessionals) *(Educator)*	*Training amount:* 4 h Support available via email	The aim of this study, therefore, was to: 1) evaluate the feedback provided from participants of a father-focused antenatal breastfeeding class (FFAB class); and 2) explore the motivations and experiences of the volunteer male peer facilitators who were trained to deliver the FFAB class.	*Methodology:* mixed methods, including a qualitative descriptive study *Data collection:* semi-structured interviews and open-ended questionnaires *Data analysis: thematic analysis*	*Sample size:* 14 *Characteristics:* not reported
McLeish and Redshaw (65) UK	*Purpose: nine different peer support interventions to support the transition to parenthood* Mothers during pregnancy and up to the infant's second birthday. “Mothers were from Black and minority ethnic communities, were asylum seekers and refugees, were young, were in prison, had very complex needs, were living with HIV, had mental health problems, or lived in a particular area”	*Delivery style:* home visiting, telephone support and group sessions *(In person one to one supporter, group supporter and remote supporter)* *Duration/frequency:* varied across interventions, ranging from pregnancy up to 2 years postpartum	*Intervention setting:* Various third sector organizations *Funding:* Chairty or public funds	*Title:* Volunteers (Mothers from the local community with experiences in common with the women they were supporting.) *(Peer volunteers)*	Active listening, providing information about pregnancy, birth and parenting, about the English healthcare system and other official systems; signposting to local services in the area. In most of the projects, the volunteers also offered material and practical support. (i.e., providing mothers with second-hand baby clothes and equipment, accompanying them to medical appointments) *(Educator, provider of emotional support, provider of practical support, connector to further support)*	*Training amount: Ranging between 8 and 72 h* Regular supervision provided	To explore experiences and perceptions of giving and receiving voluntary peer support during pregnancy and early parenthood in England, with a particular focus on disadvantaged women.	*Methodology:* Qualitative descriptive study theoretically informed by phenomenological social psychology *Data collection:* Semi-structured interviews *Data analysis:* Thematic analysis	*Sample size:* 47 *Age:* 22 years to 50s *Ethnicity:* White, *n* = 23, Black, *n* = 14, Asian, *n* = 9, Mixed race, *n* = 1. *Number of children:* range 1 and 8 children (median 2) Range from young mothers who had never worked to professional women in their 40s and 50s with adult children Sixteen volunteers spoke English as an additional language
McLeish and Redshaw (32) UK						*Training amount:* 36 h Regular supervision provided	To understand the impact and dynamics of peer support from the perspectives of those giving and those receiving support.		*Sample size:* 6 (subsample of sample reported in McLeish and Redshaw (65)) Not reported separate to non-volunteer study participants
McLeish and Redshaw (80) UK						*Training amount: ranging between 8 and 72 h* regular supervision provided	To understand the volunteers' experiences of supporting + very disadvantaged mothers.		*Sample size:* 38 (subsample of sample reported in McLeish and Redshaw (65)) *Age:* ranged from 22 years to women in their fifties) *Ethnicity:* White, *n* = 19, Black, *n* = 11, Asian, *n* =7, Mixed race, *n* = 1.
McLeish and Redshaw (64) UK	*Name:* community Doula projects *Purpose:* provide peer support, emotional support, and labor support Mothers during pregnancy and the postnatal period. Women with no partner or whose partner was unable to be present at birth, women with additional vulnerabilities (e.g., social isolation, poor mental health, domestic violence, or recent migration”	*Delivery style:* home visiting and telephone support *(In-person one-to-one supporter and remote supporter)* *Duration/frequency:* weekly support	*Intervention setting:* Provided by three community third sector organizations *Funding:* Not specified	*Title:* Doula (local community members with specific doula training) *(Non-peer volunteers)*	To provide social support, including emotional, informational, affirmational and instrumental support *(educator, provider of emotional support, provider of appraisal support, provider of practical support)*	*Training amount:* between 75 and 90 h regular ongoing supervision	The study focuses on volunteer doulas and disadvantaged mothers' understanding and experience of the community doula role during labor and birth in England, and how that interrelates with their understanding and experience of the midwife's role.	*Methodology:* qualitative descriptive study theoretically informed by phenomenological social psychology *Data collection:* semi-structured interviews *Data analysis:* thematic analysis	*Sample size: 19* *Age:* ranged from early twenties to mid-sixties, with the majority being in their thirties All but one had children, and three were grandmothers *Employment:* in paid work, *N* = 14 Full-time mothers, *N* = 3 Student, *N* = 1 Retired, *N* = 1 *Ethnicity: White British, n = 14, Asian or British Asian, n =5*. *Time in volunteer role:* range, 4 months and 6 years (mean, 3.2 years) *Number of births attended:* 1–2 births *N* = 6 3–4 births *N* = 3 6–8 births *N* = 5 over 10 births *N* = 4 0 births *N* = 1
McLeish and Redshaw (72) UK							To explore how the antenatal and postnatal role of the community doula is experienced and understood by the volunteer doulas and the disadvantaged women who they support.		
McLeish et al. (66) UK	*Name:* parents in Mind *Purpose:* to improve mental wellbeing of pregnant women and mothers *Target population:* pregnant women and mothers with a child under two years of age who self-define to have mid-to-moderate mental health difficulties.	*Delivery style:* in person face to face or in groups *(One-to-one in-person supporter and group supporter)* *Duration/frequency:* no limit on the number of sessions in some areas, and some areas encouraged women to move on after 8 sessions.	*Intervention setting:* third sector community-based programme provided nationally *Funding:* Not reported	*Title:* Peer volunteers Local mothers with past experience of mental health difficulties who are currently well. *(Peer volunteers)*	Non-directive, strengths-based active listening, Sharing information, sharing ideas for self-care and parenting and signposting to services. *(Educator, provider of emotional support, connector to further support)*	*Training amount:* 24 h *Supervision:* supported by a programme manager, trainer and mental health professional	To explore how one-to-one and group perinatal mental health peer support offered by trained volunteer peer supporters works for individual mothers, and to build an evidence-based change model identifying the positive and negative mechanisms of change, the individual contextual factors that trigger those mechanisms, and the proximate outcomes that result.	*Methodology:* Realist evaluation *Data collection:* Semi-structured in-depth interviews *Data analysis:* realist coding for context-mechanism-outcome configurations	*Sample size: 27* *Sample characteristics: Age (years):* 20-30, *N* = 15 31–40, *N* = 13 41–50, *N* = 7 51+, *N* = 2 *Ethnicity:* White British, *N* = 15 White Other, *N* = 5 Asian British, *N* = 5 Black British, *N* = 1 Black Other, *N* = 1 *Past experiences of perinatal mental health conditions:* Depression, *N* = 22 Anxiety, *N* = 10 Post-traumatic stress disorder, *N* = 5 Obsessive-compulsive disorder N1 Other *N* = 2
O'Rourke et al. (73) Australia	*Name:* birth for humankind *Purpose:* to improve maternity care and outcomes through providing social, emotional and practical support *Target population:* women experiencing socioeconomic adversity (financial hardship, refugee background, homelessness or complex trauma).	*Delivery style:* in-person support *(In-person one-to-one support)* *Frequency/duration:* during labor and 2 postnatal visits.	*Intervention setting:* community organization (Birth for Humankind) *Funding:* funded through philanthropy	*Title:* Volunteer doulas (Private doula background, midwives and midwifery students and bicultural workers.) *(Non-peer volunteers)*	To provide social, emotional and practical support *(Provider of emotional support and provider of practical support)*	*Training amount:* not specified Supervision details not reported.	To test and refine initial theory around how doulas can cause a woman to feel dignified and confident	*Methodology:* realist evaluation *Data collection:* focus groups *Data analysis: iterative theory testing and refinement*	*Sample size:* 8 *Sample characteristics*: *age, ethnicity, education:* not reported Work background: private doula, *N* =2, Bicultural doula training, *N* = 3, non-practicing midwife. Student midwife, *N* = 4, other health/social care, *N* = 1 *Time in volunteering role:* Less than a year, *N* = 1 1–3 years, *N* = 2 More than 3 years, *N* = 5
O'Rourke et al. (78) Australia							To test and refine initial theories about cultural matching being best for women.		
Raine (79) UK	*Name:* sure start breastfeeding support project *Purpose:* to support mothers with breastfeeding through peer support *Target population:* mothers from disadvantaged areas during the breastfeeding period.	*Delivery style:* group sessions and telephone support *(Group supporter and remote supporter)* *Duration/frequency:* weekly sessions plus telephone support	*Intervention setting:* sure start in a disadvantaged community *Funding:* not reported	*Title:* Breastfeeding supporters (Women who have successfully breastfed) *(Peer volunteers)*	Increase knowledge and awareness of breastfeeding benefits *(Educator)*	*Training amount:* 24 h Access to an out-of-hours helpline	To outline the rationale for establishing a peer support intervention to encourage breastfeeding and to explore the advantages of partnership working between professionals and volunteers. To explore perceptions of project coordinators, midwives, health visitors, breastfeeding supporters (volunteers) and breastfeeding mothers who had contacted the service.	*Methodology:* not specified *Data collection:* semi-structured interviews *Data analysis:* grounded theory	*Sample size:* 6 *Sample characteristics:* not reported
Richards and Lanning (58) USA	*Name:* birth partners *Purpose:* support women having a cesarean birth *Target population:* mothers only during labor and shortly after childbirth	*Delivery style:* support in the hospital setting *(In-person one-to-one supporter)* *Duration/frequency:* one-off at birth	*Intervention setting:* public academic tertiary health center *Funding:* not reported	*Title:* Doula *(Non-peer volunteers)*	Support clients' physical and emotional needs during cesarean, support skin-to-skin contact and breastfeeding *(Provider of emotional support and provider of practical support)*	*Training amount:* trained (amount varied) *Supervision:* not reported	To explore the experiences of volunteer doulas who provide support to women during cesarean birth.	*Methodology:* not specified *Data collection:* semi-structures interviews *Data analysis:* content analysis	*Sample size:* 9 *Age:* range 21 to 67 years *Education:* most had a bachelor's degree *Time in doula role:* 1 year, *N* = 1 2 years, *N* = 1 3 years, *N* = 2
Shorey et al. (59) Singapore	*Name:* not reported *Purpose:* to support the mental wellbeing of mothers *Target population:* mothers at risk of postnatal depression (score above 9 on Edinburgh Postnatal Depression Scale	*Delivery style:* phone calls, texts, WhatsApp *(Remote supporter)* *Duration: minimum once a week contact for a minimum of a month*	*Intervention setting:* technology-based peer support intervention delivered as part of a research grant *Funding:* National University Health System Collaborative Clinician Research Grant	*Title:* Peer volunteer Mothers who had recovered from postnatal depression *(Peer volunteers)*	Provide peer support, share experience, support based on the needs of the mother, making referrals when necessary for further support *(Provision of emotional support, connector to further support)*	*Training amount:* 4 h training session *Supervision:* Support available as required	To explore perceptions of a technology-based peer support intervention.	*Methodology:* qualitative descriptive study *Data collection:* qualitative interviews *Data analysis:* thematic analysis	*Sample size:* 19 *Age:* range, 25–54 years *Ethnicity:* Chinese; (*N* = 17), Malay; (*N* = 1), Indian; (*N* = 1) *Household income (monthly):* < $3,000 (*N* = 3), $3,000-5,000, (*N* = 4), >$5,000, (*N* = 7)
Shorey et al. (60) Singapore	*Name:* supportive Parenting App intervention *Purpose:* to improve social support, mental wellbeing, parental bonding and satisfaction to prevent postnatal depression *Target population:* parents recruited during pregnancy from two public health care institutions.	*Delivery style:* digital app *(Remote supporter)* *Duration/frequency: ad hoc* support from 24 weeks pregnant to 6 months postpartum	*Organization setting:* a digital app developed by a multidisciplinary research team. *Funding:* part of a research project funded by the Ministry of Health.	*Title:* Peer volunteers (Experienced mothers matched with participants based on demographic profiles) *(Peer volunteers)*	To provide informational, emotional, and affirmational support *(Educator, provider of emotional support, provider of affirmational support)*	*Training amount:* half a day *Supervision:* access to 24-h hotline for support from the research team	To explore the perceptions of peer volunteers and identify areas of improvement for the intervention.	*Methodology:* qualitative descriptive design *Data collection:* semi-structured interviews *Data analysis:* thematic analysis	*Sample size:* 18 *Age:* average, 46 years, range, 23–63 years. *Ethnicity:* Chinese, *n* = 17, Malay, *n* = 1 *Marital status:* married, *N* = 13 single, *N* = 2 divorced, *N* = 3
Spiby et al. (63) UK	*Name:* not specified *Purpose*: to provide birth support and to enhance support and improve uptake of health and social services for disadvantaged childbearing women *Target population:* mothers during pregnancy and shortly after birth. Women who “were isolated and unsupported, had physical or mental health problems, had challenging social circumstances, had particular fears about giving birth, including negative previous experiences of childbearing.”	*Delivery style:* home visiting and support in hospital setting *(In person one to one supporter)* *Duration/frequency:* from 6 months pregnant to 6 weeks postpartum	*Intervention setting:* provided across five NHS or third sector organizations *Funding:* local authority and/or Primary Care Trust	*Title:* doula *(Non-peer volunteers)*	Provide emotional support, information and physical support, birth support, accompanying appointments, going on trips, giving information, breastfeeding support *(Educator, provider of emotional support, provider of practical support)*	*Training amount:* amount not reported Mentored throughout the process of supporting a mother	To understand the motivation and experiences of volunteer doulas who have been trained to support women during pregnancy, birth and the postnatal period. Additional objectives of the broader study included exploring the implications of the service for the NHS, the health and psychosocial impacts for women, and the process of implementing and sustaining the service.	*Methodology:* mixed methods study with a realistic evaluation exploring experiences of volunteers *Data collection:* initial focus groups to guide future data collection methods. Open-ended questionnaires with some follow-up semi-structured interviews *Data analysis:* content analysis	*Sample size:* 89 *Age:* under 30, *n* = 20 30–39, *n* = 42, 40 and over, *n* = 27 *Ethnicity:* white, *n* = 71 Asian/British, Asian, *n* = 11 Black/Black, British, *n* = 5 mixed race, *n* = 1 others, *n* = 1 *Employment:* employed, *N* = 41 full-time employed, *N* = 16 part-time employed, *N* = 25 Studying or ‘other major demands on their time' *N* = 13
Taggart et al. (81) Australia	*Name:* Sutherland family network *Purpose: support families identified to be facing additional stressors* *Target population:* mothers with social or geographical isolation, socio-economic disadvantage, poor health, disability, depression or low self-esteem.	*Delivery style:* Home visiting (*In-person one-to-one supporter*) *Duration/frequency:* not reported.	*Intervention setting:* not specified *Funding:* initially through local area health budget and then sustained through Rotary's efforts.	*Title:* volunteer (Mixed experiences of mothering and women who wanted to help in the community) *(non-peer volunteers)*	Support is defined within the programme as befriending, listening, and helping with the day-to-day practical skills of mothering. It also includes liaising with other community groups and agencies, as necessary. *(Provider of emotional support, provider of practical support and connector to further support)*	*Training amount:* amount not reported Supervised, no further details provided.	The study is based on a description of the experiences of women involved in the Sutherland Family Network home-visiting project (volunteers and mothers).	*Methodology:* phenomenology *Data collection:* semi-structured interviews and focus groups (observations not reported in findings) *Data analysis:* volunteer discussions were analyzed into thematic descriptions, independent of the questions asked.	*Sample size:* 10 *sample characteristics:* not reported

**Figure 1:**
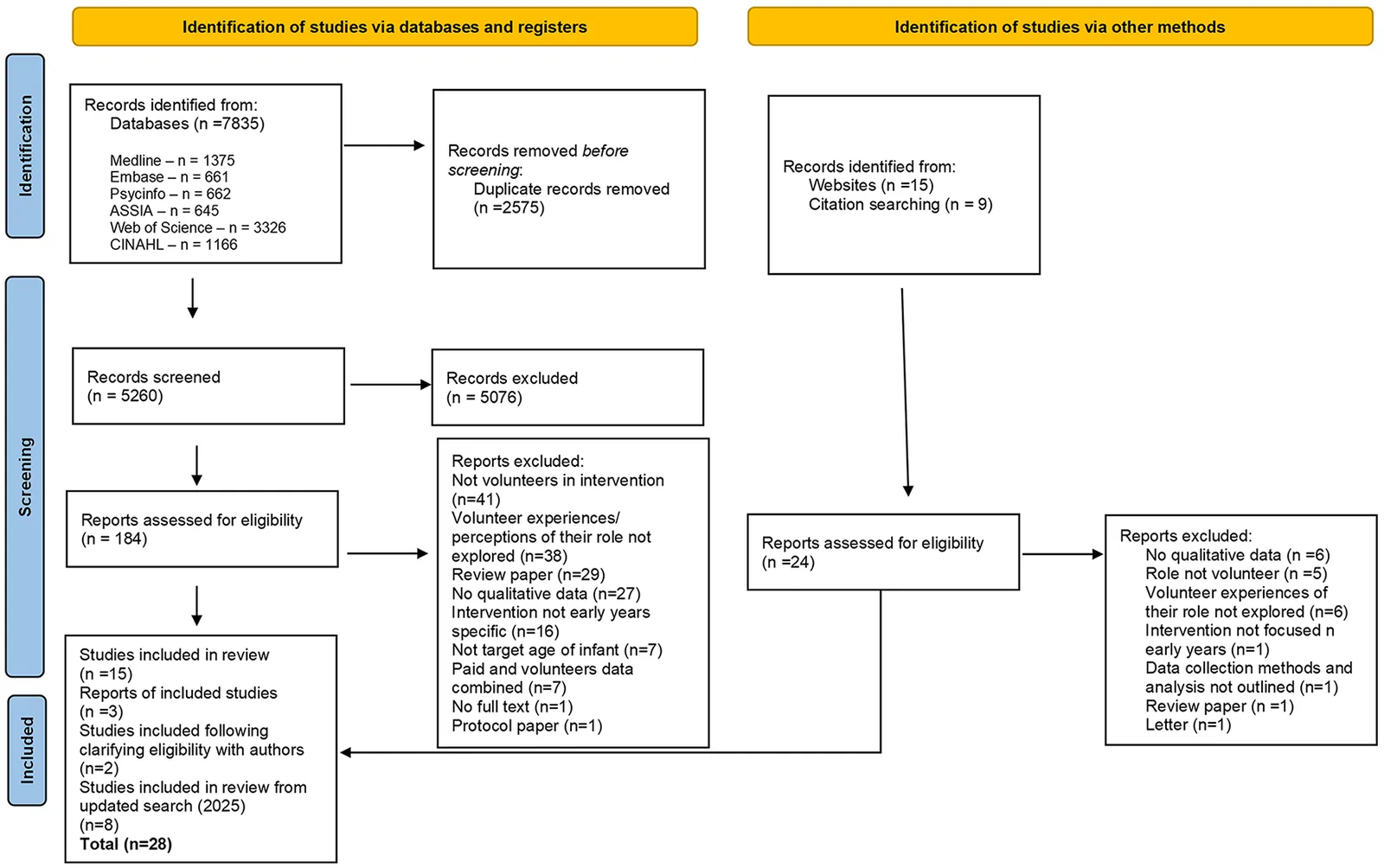
PRISMA flow diagram of included studies. From: Page et al. (104). For more information, visit: http://www.prisma-statement.org/.

### 3.1 Characteristics of included studies

Details of interventions and study designs are reported in Table 1. Studies were conducted in the UK (*N* = 15), Australia (*N* = 9), the United States (*N* = 2) (57, 58), and Singapore (*N* = 2) (59, 60). A total of 23 articles reported findings from studies with qualitative designs only, with the remaining five articles (33, 59, 61–63) drawing on qualitative and quantitative methods. Studies, where reported, were described as using a qualitative descriptive design (*n* = 11), were informed by phenomenological social psychology (*n* = 8) (32, 64–70), and were conducted from a realist evaluation perspective (*n* = 4) (11, 71–73). Data were collected through semi-structured interviews (*N* = 16), focus groups (*N* = 7), open-ended questionnaires (*N* = 3) (62, 63, 74), in-depth interviews (33, 59), or logbooks (75, 76). Data were most commonly analyzed using thematic analysis (*N* = 15), qualitative content analysis (*N* = 4) (11, 57, 58, 63).

### 3.2 Characteristics of participants

A total of 387 volunteer participants were included across studies (range: 6–89). Eighteen studies provided some detail on the characteristics of volunteer participants. The age of volunteers ranged from 20 to 60 years. Education levels were described in only three studies (58, 74, 77) with participants most commonly educated to a degree level or above. Four UK studies reported that the majority of participants described themselves as White British (61, 63, 64, 75), and participants within the two studies conducted in Singapore mostly described themselves as Chinese (59, 60). The studies reported by McLeish and Redshaw (64, 65) and McLeish et al. (66) included more diverse samples, with ethnicity described as either White, Black, Asian or Mixed Race. Only one study included fathers only (62), while all remaining participants were either described as mothers, grandmothers or women. Volunteers within over half of included studies (*N* = 18) were described as “peers” with similar backgrounds or experiences to the intervention's target population (e.g., peers within postnatal support interventions having experience of postnatal depression themselves).

### 3.3 Characteristics of volunteer roles

Participants most commonly had roles as doulas (individuals who provide emotional, information and practical support during pregnancy, labor and postnatally (67) (*N* = 10), (57, 58, 63, 64, 68, 69, 71–73, 78); within interventions to support the mental wellbeing of mothers (*N* = 7) (59–61, 66, 74–76) and to support mothers to breastfeed (*N* = 5) (27, 30, 62, 77, 79). Participants were also involved in interventions to support parenting abilities (11, 33) or to support parents during the transition to parenthood (32, 65, 80, 81). The target populations of interventions varied; however, most targeted families experiencing some form of health challenge or social disadvantage (i.e., low-income families or those living in areas of deprivation). Interventions were targeted at mothers only, except studies reported by Granville et al. (33) and Shorey et al. (60) in which volunteers supported co-parenting couples (mothers, fathers, partners and other co-parents) and Kuliukas et al. (62) in which volunteers exclusively supported fathers. Participants delivered their volunteer roles most often through in-person one-to-one role (i.e., home visiting) (*N* = 16) and less commonly through remote methods (e.g., telephone support) (33, 59, 60, 74) or through group sessions only (62). Volunteers from remaining interventions delivered their roles through a combination of these delivery models (e.g., in person and remote methods) (32, 57, 61, 64–66, 72, 79, 80). The most common elements of volunteer roles were to educate (i.e., share knowledge or information to increase parent skills and abilities) (*N* = 13) or to provide emotional support (i.e., through listening to parents share their experiences or concerns) (*N* = 1). Less commonly, volunteers provided practical support (i.e., support with attending appointments) (*N* = 7), connected parents to additional services for further support (*N* = 6) or provided appraisal support (I.e., supporting self-evaluation and providing feedback) (*N* = 4). Where details were provided (*N* = 18), the amount of support ranged from a one-off group session (62) to support lasting from pregnancy to the infant being aged 2 years (65).

### 3.4 Quality appraisal

The quality of the included articles according to the CASP checklist is presented in the Supplementary File. All studies provided a clear statement of the study's aim or purpose, were suited to a qualitative methodology, and had an appropriate research design and data collection methods that suited to the purpose of the study. In addition, all but two studies (33, 81) provided a clear statement of the study findings. Only seven studies fully reported details of the relationship between the researcher and the participants, and studies reported by Granville et al. (33), Coe et al. (61), Downie et al. (11), Raine (79), and Taggart et al. (81) did not outline the process of recruitment. Ethical considerations were not fully discussed in studies reported by Carter et al. (75), Granville et al. (33), Richards et al. (58), and Taggart et al. (81). Overall, the findings of each study provided value by adding to the field, offering implications for practice, or identifying areas for further research. However, few studies identified the value of the research across all three areas.

## 4 Qualitative synthesis

The thematic synthesis yielded three analytical themes describing the experiences and perceptions of volunteers in EYIs. The analytic and descriptive themes along with example codes are presented in Table 2, and the individual studies that contributed to the development of each theme are presented in Table 3.

**Table 2:** Themes and example codes.

Analytical theme		Descriptive theme	Illustrative codes
1. Volunteers as a mechanism for change	*1.1. Developing quality relationships*	(Perceived) Beneficial traits of volunteers	*Non-judgemental, committed, peer experience, and altruistic motivations*
		Different power dynamic to professionals	*Non-statutory, non-threatening, more friendly, and trust*
		Able to create positive relationships with parents	*Building trust and rapport, befriending, emotional bond, and listening*
	*1.2. Remit of volunteer role*	Supporting mothers to be informed	*Sharing knowledge and support with medical appointments*
		Providing parent-centered support	*Tailored/focused support and supporting parents to achieve their goals*
		Encourage parent self-belief	*Empowerment, Reassurance, developing confidence, and normalizing*
		Supporting professionals	*Reduce workloads and build relationships between parent and professional*
	*1.3. Challenges of volunteers delivering roles*	Constraints of the volunteer role	*Wanting to do more and expected to do more*
		Lack of understanding of volunteer role	*By professionals, by service users and families, and by volunteers*
		Challenges working with professionals	*Not accepted by professionals and professionals feeling threatened*
		Volunteers not feeling fully capable	*Lacking confidence and feeling underprepared*
		Unstructured workloads	*Lack of rotas and lack of consistent work*
		Challenging relationships with service users	*Managing boundaries and not developing appropriate relationships*
2. Supporting volunteers to succeed		Training needs	*Thorough training, Broader knowledge, and feeling equipped*
		Recognition	*Recognition*
		Support from colleagues/peers	*Support from staff, Peer support, and Reinforcement*
3. The transformative power of volunteering for volunteers		Increased professional development Personal development	*Motivated for career development and Pathway to employment* *Satisfaction, Self-worth, Confidence, and increased awareness*
		Increased social wellbeing	*Motivated for social gains, Improved social support, and Friendships*
		Therapeutic benefits to peer volunteers	Driven by personal past peer experiences and *meaning to own experience*
		Demands on personal life	*Balancing personal commitments and time demands*
		Negative emotional impacts	*Decreased confidence, worrying about parents, and bring back bad memories*

**Table 3:**
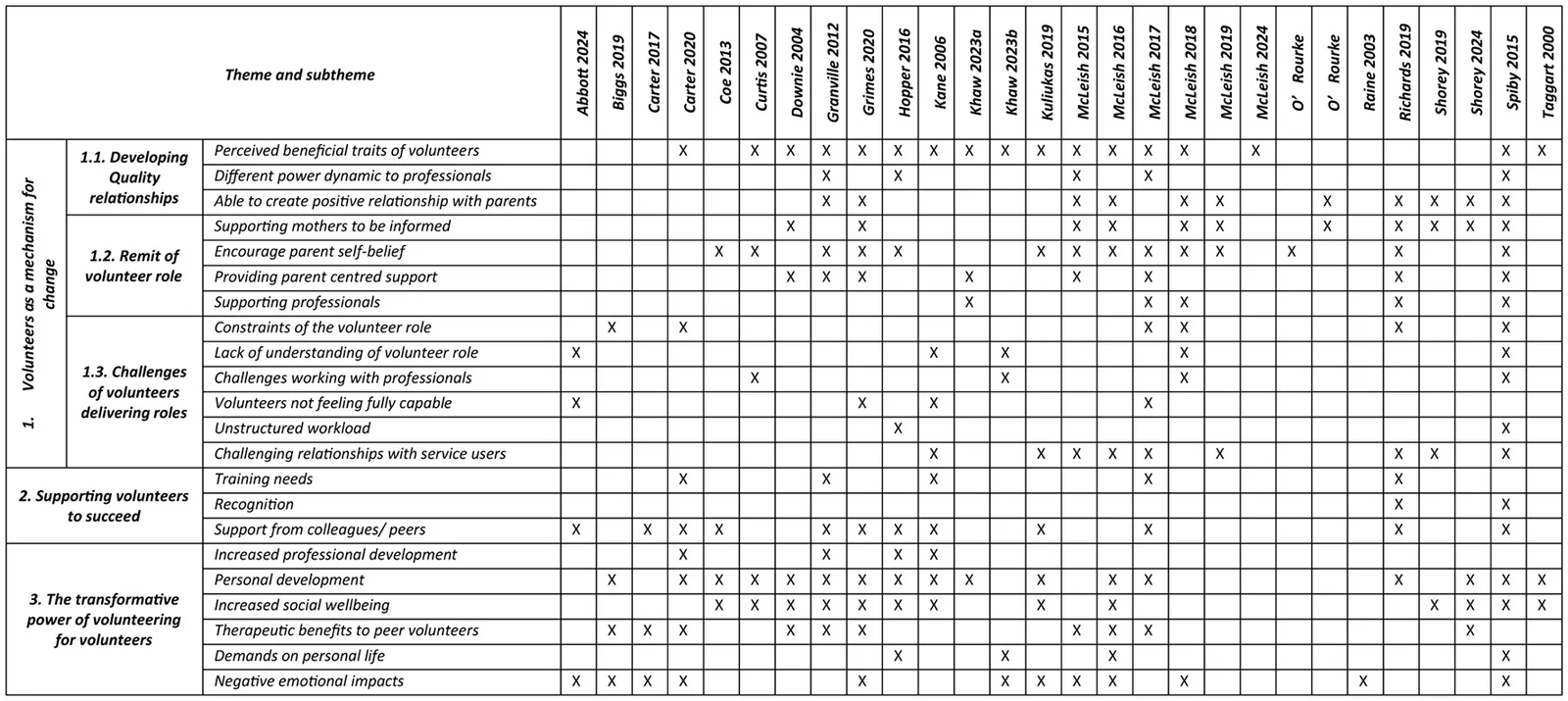
Contribution of studies to themes.

### 4.1 Analytical theme 1: volunteers as a mechanism for change

The theme of ‘volunteers as a mechanism for change' refers to the reflections from volunteers of what it is about themselves that makes them suitable to deliver the intervention and able to create the conditions to support parents to make changes to their behaviors and wellbeing. This theme comprises three sub-themes.

### 4.2 Analytical subtheme 1.1: developing quality relationships

This subtheme describes how volunteers perceive they can achieve an appropriate relationship with the parents they support, including the personal qualities volunteers have that help develop quality relationships, how they can have a different power dynamic from professionals, and how they create and maintain quality relationships with parents.

#### 4.2.1 Beneficial traits of volunteers

Volunteers described themselves as committed (27, 63, 64), patient (57), reliable (80) and non-judgemental in their support (32, 33, 63, 65, 80). Being non-judgemental was particularly important for volunteer doulas (63, 69, 71) and volunteers supporting women facing disadvantage (32, 33, 63, 65, 69, 71, 80) who explained how they were there to support mothers regardless of the mother's personal situation and would respect the parent's choices regardless of the volunteer's personal opinion.

“*I don't care about your past, I'm here for you in the ‘right here' ‘right now', in how I can help you now. My role was different from others because I didn't have an agenda and when she realized I was there just for her, that made it a very different relationship for her, and one she could talk in and confide in”*. (Quote from a volunteer working with a parent who was in the social care system (33), p. 45)

Volunteers providing their support across interventions of all different purposes (e.g., doula, breastfeeding, and parenting support) described altruistic motivations for volunteering, such as having a drive to help others (30, 33, 57, 65), having an opportunity to contribute to something positive (62), to give something back (74, 77), or to help in their local community (11, 81). Volunteers believed that giving their time up for free helped parents trust them.

*Others felt it would enhance trust because women would appreciate that the doulas were giving up their own time*. “*Because it is voluntary women can see how dedicated doulas are to their roles.”* (Quote from a Volunteer Doula (63), p. 99)

Peer volunteers across the range of EYIs (11, 30, 32, 33, 64, 66, 71, 78) viewed that having a similar lived experience meant that they could more effectively support and empathize with parents; drawing on their personal experience to provide more tailored and appropriate care. A volunteer within a peer mental wellbeing intervention also viewed that receiving support from someone with lived experience could help mothers see that it is possible to recover (66):

“*Mums do tend to like the fact that we've been where they are, but look at us now. We're dealing with it. There is a future … It helps them to see that there's light at the end of the tunnel'*. (Quote from a volunteer reflecting on having similar lived experience. (66), p. 10).

#### 4.2.2 Different power dynamic to professionals

Volunteer doulas supporting mothers with health or social problems (11) and peer volunteers supporting disadvantaged women (66) described how their personal qualities, which differed from those of professionals, helped them support parents, given how they were perceived by those they supported. Volunteers felt they were less threatening and more friendly than professionals. Volunteers reported building trust more easily because they were not seen as part of statutory services such as primary care, midwifery, or social services (11, 66). They felt this created a more equal relationship, making parents more willing to open up and share information. These reflections came from volunteers working for third sector organizations (63, 80) or a combination of third sector organizations and the National Health Service (NHS). However, there were also examples of some families being wary of volunteers and the organizations they represent (as highlighted in Section 1.3.6). Belonging to a third sector organization could explain why volunteers believe they are viewed as less statutory than health or care professionals.

*Some expressed this in terms of the relationship being non-threatening, more friendly and relaxed, or less intrusive than other services, and without any hidden agenda: “It's not statutory [so] mum can feel relaxed.”* (Quote from a Volunteer Doula (63), p. 99)

Peer volunteers supporting disadvantaged women also viewed that their roles differed, in that they did not tell parents what to do (65) and did not involve making the tougher decisions around care that professionals would normally make (33).

“*I'm not coming in with all this advice, I'm not like this health professional who's going to tell her to do this and do that with her child, I'm just there to support her in whatever decision she kind of wants to make. So I think she felt more like in control, whereas with other services, like health professionals, you don't feel in control ‘cause you feel like they're telling you what to do.”* (Quote from volunteer (65), p. 11)

#### 4.2.3 Able to create positive relationships with parents

Volunteers across all studies identified that developing a trusting and friendly relationship with the individuals they were supporting was a key and enabling element of their role (32, 59, 60, 63, 65, 72, 73, 77, 80). Volunteers described that they would create a team with parents (64) where emotional bonds were created (63, 72). However, maintaining appropriate boundaries was an important aspect of the relationship between volunteers and parents across different EYIs (33, 63, 80).

“*The first few visits are about...building up that relationship and then once you've got that trust and that bond, it's easier then to talk about the things that they maybe don't want you to talk to them about, like stopping smoking.”* (Quote from a Volunteer Doula (72), p. 6)

### 4.3 Analytical subtheme: remit of volunteer role

Across the different EYIs and volunteer roles, it was commonly identified that educating and advising, encouraging parent self-belief, providing parent-centered support, and supporting professionals were key elements of volunteer roles. Volunteers also highlighted how their roles and responsibilities were different to those of professionals.

#### 4.3.1 Supporting parents to be informed

Volunteers highlighted how an element of their role would be to share knowledge and provide relevant information, including providing specific breastfeeding advice (77) or reinforcing or translating medical advice (32, 64, 72, 80). Volunteers would also identify where parents needed further support and would share information on relevant services or directly refer parents to these services (11, 77).

“*If mum's not sure of any slang they use, we'll break that down into layman's terms for them, simplify it*”. *Sometimes during the appointment itself they had to* “*translate it into maybe simpler terms”*. (Quote from a Volunteer Doula (72), p. 7)

#### 4.3.2 Encouraging parent self-belief

Encouraging parent self-belief in their parenting abilities was an important element of volunteer roles reflected across EYIs (11, 32, 62, 63, 72). Volunteers achieved this through providing affirmation (65, 72, 77), reassurance and empowering parents to feel independent and capable (11, 30, 32, 33, 58, 61–63, 65, 72, 77, 78, 80).

“*We're validating their personal power. the validation, the acknowledgment, being by her...we see her capacity to step up into her own thoughts, that which she wants. And, yeah, then that empowers her to take that further in her life as a lived experience of, "That worked. I liked it. I can see how I might do this again”* (Quote from a volunteer doula (73), p. 15)

#### 4.3.3 Providing parent-centered support

Providing parent-centered support was central to the volunteers' approach in delivering their roles, particularly within doula interventions (58, 63, 69) interventions supporting vulnerable mothers during the transition to parenthood (65, 80) or with their parenting abilities (11, 33). Volunteers reported tailoring their support to the specific needs of parents (33, 63, 65, 80) and being able to spend dedicated quality time with parents (63). Within a breastfeeding intervention (77) and doula interventions (63, 69), support was provided over a period of months by the same volunteer to provide consistency to the service users (63, 69, 77). This was seen to help build the relationship and level of trust (77) and aid in developing a better understanding of their needs and communicating this with healthcare professionals.

“*I'm a bit of glue… because our clients are so complex they'll have…all sorts of other workers involved… a lot of those services don't communicate with each other very well… because I'm the one who's only with the client and my only job is the client…I'm the one who can kind of connect the dots for everybody else who's not communicating properly.”* (Quote from a volunteer doula who supported migrant mothers (69), p. 532)

#### 4.3.4 Supporting professionals

Volunteers with doula roles and peer volunteers supporting disadvantaged mothers in the transition to parenthood described working alongside professionals (including healthcare professionals and paid staff in EYIs). Volunteers could support the building of relationships between parents and professionals and help with relaying medical information to parents (58, 64, 80). Volunteer doulas identified that they could complement professional roles and also reduce the workload or burden of professionals by supporting the mother in the labor room. This was felt to reduce the need for midwives to check in (64, 71).

*[…] having doulas ‘takes the pressure off' overstretched midwives and could be “a great support to them”. Some participants thought that midwives might adjust their practice if a doula was present: “They don't visit quite as often…they know that…if we think there's a problem we'd call them”*. (Quote from a Volunteer Doula (64), p. 56)

### 4.4 Analytical subtheme: challenges of volunteers delivering roles

Volunteers reflected on the challenges faced in delivering their roles, which could limit their ability to achieve positive outcomes, such as roles being constrained or not understood, and challenges in achieving optimal relationships with individuals they worked alongside (i.e., professionals) or those they supported (i.e., parents).

#### 4.4.1 Constraints of the volunteer role

Volunteers intervening as doulas (72) and in interventions focused on mental wellbeing (74) or supporting vulnerable parents (32, 80) discussed how, at times, they felt parents would want support that was more appropriate to be provided by a professional. Volunteers reported they would be asked to give their opinions during labor, which was not in the remit of their role, and some volunteers reported parents wanted more than being listened to McLeish and Redshaw (72). Some volunteers expressed that they wished they could go further than the role allowed, which led to feelings of guilt when they could not address all the parents' needs.

“*I did feel quite guilty that there wasn't more that I could do...she needed so much more than what I could give*”. (Quote from a volunteer supporting disadvantaged mothers (80), p. 40)

#### 4.4.2 Lack of understanding of the volunteer role

Across many of the studies reporting on the experiences of volunteer doulas, it was identified that volunteers faced challenges due to service users, their partners, and healthcare professionals not understanding the role of a volunteer doula (57, 63, 64, 71). There were also examples where volunteers themselves did not fully understand the limits of the support they could provide (75). Volunteer doulas reflected on how there could be confusion around what the role of a doula was, in contrast to the roles of professionals during pregnancy and labor, which could lead to the doula support not being taken up. A lack of understanding of the volunteer doula role was also attributed to confusion between the role of paid and volunteer doulas (57). Those paying for the service of a doula would have prior understanding of the role and enter a “contractual relationship,” whereas individuals accessing volunteer doula support were often signposted or referred to the service and were therefore less knowledgeable of the service, which could limit how involved they become with the support (57).

Volunteers reported there could also be a lack of understanding of the volunteer doula role by healthcare professionals they worked alongside. Volunteers explained that midwives and doctors were not always clear about which responsibilities to share with volunteers (11, 67, 69), leading to volunteer doulas to explain their roles to professionals (11).

“*The doula and midwife may be looking at each other going*, ‘*Do you want to do that, or shall I do that?'...One of us is kind of sat twiddling our thumbs*.” (Quote from Volunteer Doula (64), p. 56)

#### 4.4.3 Challenges working with professionals

Volunteer doulas would often work with healthcare professionals as part of their role supporting mothers in labor. Volunteer doulas explained how some healthcare professionals were unwelcoming, that they ignored them, or viewed them as being “in the way” (63, 64, 71). Volunteers perceived that these negative experiences could be due to midwives feeling judged for having volunteers in the room, or that the doula's role of advocating for the mother and encouraging them to make decisions was getting in the way (64). However, volunteers expressed that this was not the case for all healthcare professionals, and that it was often a positive experience working with midwifery teams.

Within a breastfeeding intervention, some health visitors that volunteers worked alongside were not supportive of volunteer involvement, due to beliefs that it was the role of the health visitor to support breastfeeding (27). This could limit how well professionals and volunteers could work together to support mothers.

*Even when support had initially been forthcoming, some health professionals had been experienced as controlling. “The health visitor in our area was a bit territorial; helping women breastfeeding was her job”*. (Quote from a volunteer whose role was to support women breastfeed (27), p. 153)

#### 4.4.4 Volunteers not feeling fully capable

Anxiety or a lack of confidence of volunteers within breastfeeding (77) and mental wellbeing (74, 75) interventions could lead to volunteers not feeling fully capable of delivering their roles and becoming anxious they would not provide the right support or enough support to parents. Volunteers supporting families facing disadvantage discussed how gaps in their knowledge left them feeling not fully capable of providing support. Volunteers within a breastfeeding peer support intervention, who received < 1 day of training, voiced how a lack of knowledge on different cultural backgrounds and wider postpartum practices created anxiety about giving the wrong advice to mothers of different cultural backgrounds (77). It was also reported by volunteer doulas (57) and peer volunteers supporting vulnerable mothers during the transition to parenthood (80) that they would not always feel fully able to support families with more complex needs than they were anticipating (i.e., more complex socioeconomic situations). In some projects, the complexity of services users' personal situations had been underestimated, leading to volunteers wanting more training. However, the extent of training needed was unclear, with some volunteers who received a short amount of training feeling more prepared than those who had received more training (80).

For example, one volunteer who had received the longest training (72 hours) said that at the end of it.

“*I was still nervous, I was still very unsure*” (V011). *By contrast, none of the volunteers who received the shortest training (8 hours) described feeling nervous or unprepared. (Quotes from volunteers working with vulnerable and disadvantaged women reflecting on their training and preparedness for providing support* *(**80**), p. 40)*

#### 4.4.5 Unstructured workloads

Volunteer doulas (63) and peer volunteers within a breastfeeding intervention (30) described how unstructured workloads could lead to them feeling like a wasted resource, due to not supporting as many families as they anticipated. Volunteer doulas also experienced a lack of consistent work (63), whereas peer volunteers supporting mothers with breastfeeding faced challenges of not having formal rotas (30).

“*.... I'm passionate about the doula scheme and my main concern is it won't work if we don't get consistent work, there's no proper management of it, you get frustrated.”* (Quote from a Volunteer Doula (63), p. 88)

#### 4.4.6 Challenging relationships with service users

Some volunteers reported challenges in engaging with and building appropriate relationships with service users. Parents did not always trust the motives of volunteers, or their hesitance to engage was attributed to a lack of understanding of the organization that volunteers belonged to. This was particularly voiced by volunteers working for third sector organizations with disadvantaged women such as migrant women (80) or women who were involved with social services (72):

Several volunteers reported encountering mothers who suspected them of ulterior motives and whose suspicions could not be overcome, so no relationship had developed: “*Some people probably think...'Are[you] from the [government immigration agency]...Are you spying on us*?”. (Quote from a peer volunteer supporting disadvantaged women (80), p. 39)

Volunteer doulas who only provide support during the birth reported that the lack of time to build rapport with mothers could make relationship building challenging, leading to a recommendation for volunteers to meet women at an earlier appointment (58). Similar recommendations for a one-off face-to-face meeting were made by volunteers within a mental wellbeing intervention who provide their support to parents through a messaging App, to overcome the challenges they face in building rapport, possibly due to providing their support remotely (59).

Other challenging elements of the relationship between volunteers and service users were maintaining boundaries, remaining emotionally distant (65) and being non-judgemental (80). Volunteer doulas (57, 63) and volunteers working with disadvantaged mothers (32, 65, 80) experienced parents who would overstep the boundaries of the volunteer support. Delivering support through a group session rather than one-to-one could help to avoid parents becoming dependent on the volunteer (65). Some volunteers gradually cut down contact with parents and signpost them to ongoing support to facilitate the end of their relationship (62, 63, 65).

### 4.5 Analytical theme: supporting volunteers to succeed

Volunteers reflected on what they needed to succeed in their roles. Meeting training needs, recognition of volunteer roles and support from colleagues and peers were factors that could contribute to volunteers successfully delivering their roles.

### 4.6 Training needs

Volunteers with roles in mental wellbeing (74), doula (57, 58) and parenting interventions (33) and those to support the transition to parenthood (80) discussed their views on the training they received. Some viewed their training as equipping them to deliver their role (58, 74, 80) and enabled them to work with families facing a range of challenges (33, 80). Others felt that further training would help them to support more complex families (80). Volunteer doulas recommended that ongoing education was needed to be able to deliver their roles successfully (57). Wider training to support where to signpost for additional support was also recommended (33, 57).

*Whilst acknowledging the high level of training received, one volunteer recommended that there was more training for volunteers on Social Care as an area of further training development. She felt that additional training would be beneficial to give an insight into: “What social care is about, what avenues they can go down, when they take children away, why they watch families”* (Quote from volunteer who provides support to disadvantaged families (33), p. 33)

### 4.7 Recognizing volunteer roles

Volunteer doulas viewed that increased recognition of the volunteer role within healthcare settings could enable them to deliver their roles more efficiently and increase their motivations (58, 63). They suggested how organizations could do more to raise the profile of volunteer-led services with partner organizations or services, as a way of making volunteers feel more accepted.

“*…it would be nice to be more recognized for the work we do and the differences we make.”* (Quote from a volunteer doula, (63), p. 118)

### 4.8 Support from colleagues/peers

Volunteers viewed that having support from paid intervention staff could provide support understanding that they always had somebody to go to, particularly to debrief on challenging experiences (30, 33, 58, 61–63, 74, 76, 80). Volunteer doulas explained that they could experience traumatic events (including instances where mothers who were prisoners were separated from their babies) and highlighted the specific need for trauma-informed support from the team they work alongside (68).

The support received from fellow volunteers was also seen as beneficial, as it enabled to sharing advice and learning from each other's experiences. This was felt to provide confidence to volunteers in delivering their role (33, 57, 63, 80). It was recommended by volunteers who support parents facing stressors that they should have more opportunities for volunteers to come together and support each other (81).

“*Also the other volunteers, we chat to each other a lot - maybe if you just want someone's advice or their thoughts on something so you really, really are well supported. I would definitely say that there should be no volunteer who should ever feel that they don't know what to think or don't know what to do because there is always someone at the end of the phone”*. (Quote from a volunteer who provides support to disadvantaged families (33), p. 48)

### 4.9 Analytical theme: the transformative power of volunteering for volunteers

Volunteers across the studies reflected on how their volunteering experiences affected them personally, with both positive and negative outcomes. They explained how the potential for professional and personal development motivated them to volunteer. However, volunteers also acknowledged their roles could lead to some negative consequences.

### 4.10 Increased professional development

Volunteers with roles in mental wellbeing (74), parenting skills (33), breastfeeding (30) and doula interventions (57) were motivated to volunteer within EYIs to gain knowledge, experience and qualifications that could support their career development into professions such as a paid doula or midwifery. Some volunteers within a parenting intervention reported their role directly led them into paid employment or further education (11).

*While some volunteers have left the CM program and gone back into the workforce as teachers aides others have gone on to study at TAFE in courses such as computing, child and community studies and also progressed to university studies*. (Author description of theme: facilitating personal empowerment (11), p. 195)

Volunteers supporting mothers with their mental wellbeing viewed their role as having increased their confidence, skills and understanding of perinatal depression, which could be transferred to the caring professions they were already working in Biggs et al. (74). Volunteers within a parenting intervention were able to take their experience and skills into community roles, such as committee membership and promoting their roles at conferences (33).

### 4.11 Personal development

Volunteers across all EYIs gained personal satisfaction and enjoyment through supporting parents (11, 27, 30, 33, 57, 60–63, 74, 75, 77, 80, 81) and seeing an improvement in the parents' abilities following their support (30, 63). Volunteers also reflected on an increase in their own confidence (27, 33, 60, 61, 63, 74) and feeling empowered (11, 32, 58). Those who worked alongside healthcare professionals in breastfeeding (27) or doula (63) roles also viewed they were more confident in talking to people, accessing services and challenging authority.

*This general improvement in peer supporters' self-esteem and interpersonal skills, and their greater assertiveness, was observed both by volunteers and health professionals. Individual volunteers felt more able to constructively challenge authority. They felt that they were more confident and tolerant, less judgemental and had better listening skills. Moreover, these changes were not confined to their voluntary work but extended beyond this, into other areas of their lives*. (Author description about volunteers who provide breastfeeding support (27), p. 151)

Volunteers who supported mothers experiencing disadvantage (69), vulnerability due to living with human immunodeficiency virus (HIV) (32), facing health or social problems (63) or were isolated or disadvantaged mothers (81) also reflected on how their experience changed their perspectives on how other people lived and developed a better understanding of different cultures.

*They also believed that visits had broadened their personal outlook on the community, that they had become aware of other family circumstances, cultures and situations*. (Author description of volunteers changed perspectives (70), p. 4)

### 4.12 Increased social wellbeing

Volunteers who delivered their role in person, either on a one-to-one basis or in a group setting, reported that their motivation for volunteering was to gain social skills and be more active in their communities (11, 27, 30, 33, 61, 81). Many gained a sense of belonging through the network of volunteers, with whom they also developed friendships (27, 57, 59, 62, 63, 77). Where volunteers delivered their roles remotely, they benefitted from social support through relationships built during initial training (60). Rewarding relationships were also developed with the parents they supported (11, 32, 62, 63, 81).

*For volunteers, involvement in the peer-support scheme helped both to combat social isolation and to ease pressures in their relationships with their partners*. (Author description of theme: benefits for volunteers of working with Breastfriends Breastfeeding Service (27), p. 151)

### 4.13 Therapeutic benefits to peer volunteers

Peer volunteering within EYIs that draw on personal experiences was viewed to have a cathartic effect on the volunteers. Providing support to parents who are in similar situations to what they had been previously would help volunteers understand their past experiences of mental ill health better (60, 74) and come to terms with negative experiences of breastfeeding (77) or living with HIV as a mother (32). Volunteers gained satisfaction at how their personal experience could be used in a positive way (74). Peer experiences generated empathy, which motivated volunteers to support parents in similar situations.

“[…] personally satisfying knowing that my personal experiences have positively contributed to a callers recovery in some small way. Some good has come of my experience and I derive a sense of satisfaction from this knowledge.” (Quote from volunteer who provides mental wellbeing peer support (74), p. 487)

Supporting women who are currently in a situation that is similar to those peer volunteers had been in previously provided the opportunity for peer volunteers to see how far they had come in their own recovery (75, 76).

“*Providing the support to my lady made me recognize my own strength, how ill I had actually been but how hugely different I now felt. It was therapy for me too!” (Sharon) (*Quote from a peer volunteer reflecting on her experience of supporting women with antenatal depression following their own personal experience (76), p. 265)

### 4.14 Demands on personal life

Volunteers reflected on how taking on volunteer roles within EYIs could place demands on their personal lives. Volunteer doulas voiced that the roles could take time up, causing pressure when they had competing responsibilities at home (57, 63). In addition, volunteers who delivered group sessions in the evening (62) or provided support through the night (80) would have less time to spend with their own families.

*Throughout the discussions, a repeated issue noted was balancing the passion for being a doula and desire to help others with the personal demands of the doulas' own families*. (Author description of theme: internal dilemmas (57), p. 28)

### 4.15 Negative emotional impacts

Volunteers within a mental wellbeing intervention (74) and volunteers supporting disadvantaged mothers (32, 72, 80) reflected on how they could be exposed to distressing situations, causing them to worry about the parents they support and potentially having a negative emotional impact on the volunteers. In particular, volunteer doulas who support mothers in prison felt that the pain of the mother would transfer to the volunteer when witnessing mothers be separated from their babies (68).

*The transference of painful emotions was a phenomenon described by many of the birth supporters who demonstrated empathy with the women but found caring in these circumstances complex and unforgettable*. (Author description of the transference of pain to volunteers (68), p. 4)

Volunteers who provide their support over a period of months could experience negative emotions when ending relationships with parents (11, 64, 79, 80, 82). Those who delivered breastfeeding education classes to fathers expressed feeling a sense of loss when the service they were involved in was no longer running (62). Negative impacts on volunteers were also attributed to feeling unwelcome within a doula role (63) or isolated within their role of supporting women with breastfeeding (79).

“*Finances, lack of communication, felt uncomfortable in office and meetings, didn't feel like I was accepted.”* (Quote from a volunteer doula reflecting on the challenges of their role (63), p. 116)

Although the perceived impact of drawing on past personal experiences was viewed positively, it was highlighted by a peer volunteer who supported mothers also living with HIV and volunteers providing support to mothers within mental wellbeing interventions (75, 76) that they experienced negative emotional impacts due to be reminded of past negative experiences (32).

*[…] mothers' stories could bring back memories of their own experiences:* “*They are always at a very low point and it just brings back all that I felt*” (Quote from volunteer who provides peer support to mothers living with HIV (32), p. 6).

## 5 Discussion

This review has synthesized the views and experiences of volunteers across different EYIs. These findings have provided new knowledge of how and why volunteers can successfully support parents in the first 1,001 days, contribute to positive outcomes for parents and infants, and what they need from the service to ensure this. The findings have also identified how volunteers can be broader beneficiaries, highlighting the wider social value created through volunteers delivering EYIs.

The level of connectedness and trust between a recipient and an intervention provider can influence intervention outcomes (82, 83). The use of volunteers in health and care interventions is often considered to be less judgemental and stigmatizing, for example when working with individuals requiring mental health support (70) and when working with families alongside child protection and social care workers (84). In this review, volunteers across different types of EYIs reflected on how they could specifically facilitate the building of a trusting relationship with parents. This can be explained by social capital theory, in which people who are seen as more equal, can achieve bonding social capital (85). This can then create conditions for social support to be provided (i.e., the provision of informational, emotional, appraisal and practical support) (86). However, while volunteers have previously been shown to successfully engage with those less likely to access statutory services (10), a significant contribution of this QES is the finding that parents facing disadvantage (e.g., with insecure immigration status) did not always trust the volunteers' motives or the charitable organizations they represented. Families experiencing social and economic disadvantage are more likely to experience inequalities in their health and care (87). There is a risk that volunteer-led interventions, while addressing barriers to support for some parents, may serve to increase inequalities for certain groups of disadvantaged families. Service managers and commissioners should consider how trust and perceived safety may vary across different groups and ensure that volunteer-led services are designed to address, rather than inadvertently reinforce, existing inequalities. Providing clear information about the volunteer's role, the organization they represent, and how the service differs from statutory provision may help to build trust and increase engagement.

Many of the volunteers within studies included in this review were described as peers. Peer volunteers particularly felt they could effectively support parents through drawing on their similar lived experiences. This is consistent with previous research showing that peer supporters can draw on their personal insights and experience of methods of support worked and did not work for them to deliver more effective support (88). Shared personal experiences can improve service users' confidence in the volunteer's expertise, and support the development of trusting relationships with parents (89). The benefits of this can be explained by social comparing theory (90), which posits that comparing oneself to somebody in a better position can increase confidence in one's ability to be in a better position as well. While most peers within this review had similar lived experience to those they supported (e.g., experience of perinatal mental illness), the extent to which volunteers had similar personal characteristics (e.g., similar culture) to the parents they support is unclear. This is in part due to a lack of demographic descriptions of volunteers in the included studies in this review and less discussion of the extent to which volunteers are matched based on qualities other than similar lived experience, representing a gap in knowledge about how this might impact the success of the role.

Within interventions supporting the mental wellbeing of parents and those targeting families facing disadvantage, some volunteers viewed that there were times their role as a volunteer was not suitable. Providing clarity around the remit of the volunteer role and instances when a professional should or should not intervene has been recommended in other health service settings (91) which has been shown to help manage expectations for both the volunteer and service user and can prevent volunteer burnout (92). The findings of this review suggest that providing clarity around roles is a particularly important consideration for managers of volunteer-led EYIS, which could help safeguarding both service users and volunteers. In addition, a review of the evidence of how to support volunteers across all role types identified that appropriate training and support from paid staff can aid with setting expectations of the role and ensure volunteers feel fully equipped to deliver support (93) and have a more positive experience (91). While this QES has provided some insight into the specific training needs of volunteers within EYIs, further research exploring how to successfully manage volunteers taking on the specific roles and responsibilities within different EYIs would be beneficial for providing more tailored guidance and increasing the likelihood of intervention success.

This QES has also highlighted how volunteers were able to personally gain from delivering their roles. Career development is a common motivation for all types of volunteering (94), and some volunteers in parenting and doula interventions reported that their experiences led to employment pathways. Indeed, volunteers across EYIs of all purposes emphasized that their roles led to personal development, including increased knowledge, confidence, and self-esteem. These findings suggest that volunteering within EYIs has the potential to lead to employment by providing confidence and skills to pursue paid work. Volunteers within EYIs also gained from their roles, with increased social wellbeing. Enhanced social wellbeing is a common positive effect of volunteering (37) and a key reason that volunteering is viewed as an intervention in itself for improving the health and wellbeing of volunteers (36). Where roles were delivered remotely, volunteers valued the opportunity to meet with other volunteers during training sessions. As found in a previous review of the literature on supporting volunteer wellbeing (42), this review suggests that volunteer managers should provide opportunities for volunteers to connect.

The benefits described by volunteers align with the functional approach to volunteerism, which outlines social and career benefits as two functions or motivations that can be met through volunteering (95). The promotion of these potential benefits can support tailored recruitment of volunteers (96), while assuring benefits and preventing negative impacts can support retention of volunteers (97) which can support the sustainability of EYIs. These benefits are based on self-reported perceptions by the volunteers. The future triangulation of these findings with quantitative studies measuring the impact of volunteering on volunteers could support with determining causation.

Peer volunteers specifically gained from their roles through having the opportunity to make use of their past personal experience which led to satisfaction and a better understanding of their past experiences. Peer support models have previously found to benefit the peer provider within mental health services, by helping them with their own recovery and providing access to social support (98, 99). Within this QES, the findings of positive gains of peer support were reported by volunteers with a range of roles, highlighting the potential benefits beyond mental wellbeing interventions. However, in contrast, this QES also found that peer volunteering may also trigger negative experiences, (for example, through peer support for mothers living with HIV), or through being exposed to challenging situations (such as those supporting mothers giving birth in prison).

This review highlighted further negative impacts on volunteers within doula roles in relation to managing volunteering commitments within their personal lives, particularly where support could be required during the night. These findings are consistent with other healthcare settings where volunteers may be exposed to people experiencing hardship (40, 41). As volunteer experience and satisfaction can impact on the retention of volunteers, and therefore the sustainability of services, it is important that the negative consequences of volunteering on the volunteer are considered and addressed (100). This could suggest volunteers working with families with more challenging needs may need additional support and supervision from paid roles or volunteer support groups. Where volunteers are exposed to traumatic experiences, trauma informed care may be required.

The findings of this review support policies and programmes such as the UK Start for Life programme (101), which recommend involving volunteers in supporting families during the early years. Volunteers may be particularly well placed to overcome barriers to engagement, build trust and draw on their personal experiences to provide relatable and accessible support. The review also highlights potential benefits for volunteers themselves, suggesting that volunteer-led EYIs may generate wider social value beyond benefits to families and services. These potential benefits should therefore be considered when making funding and commissioning decisions. However, services need to be designed and managed in ways that enable volunteers to benefit meaningfully from their roles. This requires adequate training, supervision and opportunities for personal development, all of which may require additional time and resources from paid staff. Volunteer-led EYIs should therefore be adequately funded to ensure that staff have sufficient capacity to support volunteers both to deliver their roles effectively and to gain personally from their involvement. Future studies exploring the cost-effectiveness of different types of volunteer-led EYIs and longitudinal studies exploring the longer-term consequences of volunteering within EYIs and how these impacts on retention of volunteers could strengthen the evidence base informing policy and practice.

### 5.1 Strengths and limitations

This review has provided an understanding of the experiences of volunteers within EYIs based on the views of a large number of volunteers. A thorough search strategy was developed, which also included gray literature sources, that has ensured that the findings of the review are based on a wide range of up-to-date evidence base. In addition, the quality appraisal conducted as part of the review highlighted that the overall quality of included studies was relatively high. However, individual studies within the review did not always provide information about the demographics of the volunteers included in their research. This has implications for determining whether the experiences of volunteers highlighted in this review are reflective of volunteers of different backgrounds.

While this review has provided some insight into how volunteers within EYIs are personally impacted from their roles, this was not the main focus of many of the included studies. This may provide less confidence that the review findings reflect all potential consequences of volunteering on the volunteer. In addition, the review did not identify volunteer views on the extent to which volunteers should be relied upon to deliver early years interventions, as opposed to the state funding paid staff to provide services. Future studies focusing on the impact on the volunteer including the negative consequences of choosing a volunteer model would contribute to a more balanced understanding of whether volunteers should be relied upon to provide EYIs.

The transferability of the findings may also be limited. First, the limited reporting of participant demographics across the included studies makes it difficult to explore how volunteer experiences may vary according to personal characteristics. Future research should explore who is accessing volunteer opportunities within EYIs and how experiences may differ to understand how equitable volunteer opportunities are within this setting.

Second, although studies from any high-income country were eligible for inclusion, the evidence was predominantly drawn from the UK and Australia, potentially limiting the geographical transferability of the findings. This may reflect the greater prevalence of, or evaluation of, volunteer-led EYIs in these countries; however, it also means that the findings may be most applicable to the UK and Australian contexts. Differences in healthcare systems, the role of voluntary and community organizations, and volunteering cultures may influence how volunteer-led EYIs are implemented, how volunteers are perceived and trusted, and how families access and experience support [e.g., the established involvement of voluntary and community organizations alongside health and early years services in the UK (102) and Australia (103)]. These contextual differences should therefore be considered when applying the findings to other high-income country settings.

Finally, while all included volunteer-led EYIs supported families during the first 1,001 days, there was considerable variation in how services were delivered. Where findings were specific to particular intervention types or volunteer roles, this has been highlighted. However, variation in the focus, level of detail and amount of data reported across studies meant there was not always sufficient comparable evidence to determine whether volunteer experiences differed according to intervention type or volunteer role. Further research, and potentially an updated review incorporating a larger and more consistent evidence base, may enable more specific comparisons between intervention types.

## 6 Conclusion

This review suggests that volunteers saw their non-professional status as a unique benefit, leading to a positive experience for the parents they supported. Volunteers are suited to, and should therefore be considered to deliver EYIs, where their personal experiences, unique abilities to create a trusting relationship or avoid a more “threatening” statutory figure are instrumental in achieving the desired outcome of the intervention. However, the remit of the volunteer role should be clear to both volunteers and service users and appropriate training and supervision provided to ensure volunteers feel capable of their role. Further research on the extent volunteers should be similar to those they support and within which EYIs is of importance is needed.

This review also identified volunteers can be direct beneficiaries of EYIs. Volunteers gained skills and experience that support personal and professional development. An increase in wellbeing was also attributed through having the opportunity to work through past negative experiences and becoming more socially connected with families and fellow volunteers. These potential gains should be promoted and supported by those designing and managing EYIs to aid recruitment and retention of suitable volunteers and maximize the value of the service. However, lack of demographic data of volunteer participants within this review warrants the future research to be able to explore if experiences and impacts of volunteering in EYIs is the same for volunteers with different personal characteristics. Further exploration of potential negative impact on the volunteer is recommended to provide a more balanced evidence base to support decision making around including volunteers in the delivery of EYIs.
