# Supplementary material for: The perceived impact of early years interventions being delivered by volunteers: a qualitative evidence synthesis of volunteer experiences
Source: Front Public Health. 2026 Oct 13;14:1933122. doi: 10.3389/fpubh.2026.1933122 (PMC13712161; doi:10.3389/fpubh.2026.1933122)
Supplement: Supplementary file 1 [file Data_Sheet_1.pdf]

Contents

Supplement 1 – Example academic database search results 2
Supplement 2 – Quality appraisal results 6
Supplement 3 – ENTREQ reporting checklist 9
Supplement 4 – PRISMA reporting checklist 12

Supplement 1 – Example academic database search results

Ovid MEDLINE(R) and In-Process, In-Data-Review & Other Non-Indexed Citations <1946 to February 03, 2022>

Searched 04.02.2022

1	Volunteer*.ti,ab.	203240
2	Volunteers/	10413
3	Unpaid worker*.ti,ab.	16
4	Unpaid staff.ti,ab.	5
5	Lay support.ti,ab.	56
6	Peer support*.ti,ab.	5319
7	Peer to peer.ti,ab.	1667
8	Parent to parent.ti,ab.	474
9	Community champion*.ti,ab.	31
10	Befriender*.ti,ab.	53
11	Peer educator*.ti,ab.	794
12	peer counsel*.ti,ab.	587
13	peer*.ti,ab.	106067
14	Mentor*.ti,ab.	18240
15	Charity worker*.ti,ab.	8
16	Voluntary worker*.ti,ab.	74
17	Voluntary staff.ti,ab.	17
18	Doula*.ti,ab.	410
19	Experience*.ti,ab.	1184581
20	Perception*.ti,ab.	280605
21	Opinion*.ti,ab.	114334
22	Attitude*.ti,ab.	164650
23	Motivat*.ti,ab.	148395
24	Benefit*.ti,ab.	757437
25	Challenge*.ti,ab.	762364
26	Advantage*.ti,ab.	465531
27	Disadvantage*.ti,ab.	84175

28	View*.ti,ab.	505058
29	Early years.ti,ab.	4270
30	First 1000 days.ti,ab.	278
31	First 1001 days.ti,ab.	2
32	Early childhood.ti,ab.	28632
33	Infant.ti,ab.	176116
34	Infant/	837417
35	Infancy.ti,ab.	54908
36	Baby.ti,ab.	40456
37	Baby.ti,ab.	40456
38	Infancy.ti,ab.	54908
39	Baby.ti,ab.	40456
40	Babies.ti,ab.	38983
41	Toddler*.ti,ab.	12401
42	First years.ti,ab.	4210
43	P*diatric.ti,ab.	352847
44	Antenatal.ti,ab.	39142
45	Postnatal.ti,ab.	110561
46	Neonatal.ti,ab.	219106
47	New*born.ti,ab.	129369
48	Mother*.ti,ab.	236219
49	Parent*.ti,ab.	444583
50	Carer*.ti,ab.	15889
51	Primary carer*.ti,ab.	271
52	Family.ti,ab.	850539
53	Families.ti,ab.	261545
54	Maternity.ti,ab.	22897
55	Maternal.ti,ab.	274360
56	Father.ti,ab.	25362
57	Paternal.ti,ab.	24302
58	Pregnancy.ti,ab.	408446

59	Pregnant.ti,ab.	196137
60	Expectant mothers.ti,ab.	1122
61	Intervention.ti,ab.	676554
62	Service*.ti,ab.	563620
63	Support.ti,ab.	1097058
64	Program*.ti,ab.	953835
65	Parenting.ti,ab.	20948
66	Parenting skills.ti,ab.	906
67	Parent* confidence.ti,ab.	321
68	Parent* satisfaction.ti,ab.	1933
69	Wellbeing.ti,ab.	20652
70	Emotional wellbeing.ti,ab.	936
71	Mental wellbeing.ti,ab.	1076
72	Psychological wellbeing.ti,ab.	1734
73	Mental Health/	50352
74	Attachment.ti,ab.	111366
75	Relationship.ti,ab.	1112469
76	Breast Feeding/	41175
77	Breastfeed*.ti,ab.	28638
78	Social development.ti,ab.	3444
79	Emotional development.ti,ab.	1780
80	Behavi* development.ti,ab.	2170
81	Child development.ti,ab.	6805
82	interview*.ti,ab.	395299
83	Interview/	30121
84	experience*.tw.	1184581
85	qualitative.ti,ab.	261172
86	Mixed method*.ti,ab.	28905
87	Multimethod*.ti,ab.	2093
88	Multiple method*.ti,ab.	3321

89 1 or 2 or 3 or 4 or 5 or 6 or 7 or 8 or 9 or 10 or 11 or 12 or 13 or 14 or 15 or 16 or 17 or 18 329453

90 19 or 20 or 21 or 22 or 23 or 24 or 25 or 26 or 27 or 28 3778470

91 29 or 30 or 31 or 32 or 33 or 34 or 35 or 36 or 37 or 38 or 39 or 40 or 41 or 42 or 43 or 44 or 45 or 46 or 47 or 48 or 49 or 50 or 51 or 52 or 53 or 54 or 55 or 56 or 57 or 58 or 59 or 60 3320610

92 61 or 62 or 63 or 64 2846813

93 65 or 66 or 67 or 68 or 69 or 70 or 71 or 72 or 73 or 74 or 75 or 76 or 77 or 78 or 79 or 80 or 81 1352854

94 82 or 83 or 84 or 85 or 86 or 87 or 88 1646257

95 89 and 90 and 91 and 92 and 93 and 94 1483

96 limit 95 to yr="2000 -Current" 1375

Supplement 2 – Quality appraisal results

	**1. Was there a clear statement of the aims of the research?**	**2. Is a qualitative methodology appropriate?**	**3. Was the research design appropriate to address the aims of the research?**	**4. Was the recruitment strategy appropriate to the aims of the research?**	**5. Was the data collected in a way that addressed the research issue?**	**6. Has the relationship between researcher and participants been adequately considered?**	**7. Have ethical issues been taken into consideration?**	**8. Was the data analysis sufficiently rigorous?**	**9. Is there a clear statement of findings?**	**How valuable is the research?**
Abbott, 2024	Yes	Yes	Yes	Yes	Yes	Yes	Yes	Yes	Yes	Implications for practice and policy clearly outlined with practical recommendations provided.
Biggs,2019	Yes	Yes	Yes	Yes	Yes	Yes	Yes	Yes	Yes	Implications of findings and areas for future research not clearly stated.
Carter 2017	Yes	Yes	Yes	Yes	Yes	No	Can't tell	Can't tell	Yes	Areas for future research provided. How findings align with other relevant research discussed.
Carter 2020	Yes	Yes	Yes	Yes	Can't tell	No	Yes	Can't tell	Yes	The study adds to existing knowledge and provides recommendations for practice.
Coe, 2013	Yes	Yes	Yes	Can't tell	Yes	Can't tell	Yes	Yes	Yes	Implications of the intervention are discussed in the conclusion but not how the findings contribute to the wider literature. Areas for future research not outlined.
Curtis, 2007	Yes	Yes	Yes	Yes	Yes	No	Yes	Yes	Yes	Within the conclusion, the implications of the study findings are discussed and highlights where future research would be beneficial (i.e., conducting observations).
Downie, 2004	Yes	Yes	Yes	Can't tell	Yes	Can't tell	Yes	Yes	Yes	Findings highlight how the intervention is beneficial to volunteers, however transferability of findings and further research areas are not discussed.
Granville, 2012	Yes	Yes	Yes	No	Yes	No	No	No	No	The findings are clearly presented, however the implications of the findings are not clearly stated. It is not clear if the findings are transferable.
Grimes, 2020	Yes	Yes	Yes	Yes	Yes	No	Yes	Yes	Yes	The study provides a clear summary of what the research has found. It is highlighted where future research could be beneficial. (i.e., research translation within community settings).
Hopper, 2016	Yes	Yes	Yes	Yes	Yes	Yes	Yes	Yes	Yes	The authors outline how the study findings have implications for practice showing transferability to

										organisation where peer support is considered. Recommendations for further research is outlined.
Kane Low, 2006	Yes	Yes	Yes	Yes	Yes	Can't tell	Yes	Yes	Yes	The researchers discuss the implications the findings can have for other organisations implementing a similar intervention which therefore highlights some transferability of findings. Some discussion around future research if doula roles were to be expanded.
Khaw 2023a	Yes	Yes	Yes	Yes	Yes	Yes	Yes	Yes	Yes	Findings of this study are discussed in relation to what they add to the existing literature. Areas for future work are clearly highlighted.
Khaw 2023b	Yes	Yes	Yes	Yes	Yes	Yes	Yes	Yes	Yes	Findings of this study are discussed in relation to what they add to the existing literature. Areas for future research highlighted. Implications for practice are highlighted.
Kuliukas, 2019	Yes	Yes	Yes	Yes	Yes	No	Yes	Yes	Yes	Direction for a further study is outlined, however the study is not generalisable. However the study determined the feasibility and acceptability of engaging fathers as breastfeeding supporters.
McLeish, 2015	Yes	Yes	Yes	Yes	Yes	No	Yes	Yes	Yes	The implications of the study findings are discussed in relation to how they can be useful in similar interventions using peer support and considerations of evaluations of future peer support interventions. Areas for future research is not discussed.
McLeish, 2016	Yes	Yes	Yes	Yes	Yes	No	Yes	Yes	Yes	The findings are discussed in relation to the literature and it is highlighted where future research would be beneficial. Transferability of findings is suggested when recommendations based on the findings are made in the conclusion.
McLeish, 2017	Yes	Yes	Yes	Yes	Yes	No	Yes	Yes	Yes	Implications of findings are provide in the conclusion with recommendations for where future research would be beneficial. Transferability of findings would be to interventions delivered by volunteers supporting disadvantaged women.
McLeish, 2018	Yes	Yes	Yes	Yes	Yes	No	Yes	Yes	Yes	The study findings clearly address the objectives and therefore address the reasons why the study was conducted. There is no clear statement on the implications the findings have in practice but the conclusion states an area where future research could be needed.
McLeish, 2019	Yes	Yes	Yes	Yes	Yes	No	Yes	Yes	Yes	The study findings clearly address the objectives and therefore address the reasons why the study was conducted. There is no clear statement on the implications

										the findings have in practice but the conclusion states an area where future research could be needed.
McLeish, 2024	Yes	Yes	Yes	Yes	Yes	No	Yes	Yes	Yes	Findings of this study discussed against the wider literature. The discussion clearly states how this research adds knowledge to the field. Area for future research clearly highlighted.
O'Rourke 2022a	Yes	Yes	Yes	Yes	Yes	No	Yes	Yes	Yes	Findings are discussed within the wider literature and where these findings may be of relevance in practice highlighted at the end of the discussion. Areas for future research not clearly highlighted.
O'Rourke 2022b	Yes	Yes	Yes	Yes	Yes	No	Yes	Yes	Yes	Findings are discussed within the wider literature. Implications for the intervention are highlighted and suggestions for future research provided.
Raine, 2003	Yes	Yes	Yes	Can't tell	Yes	Can't tell	Yes	Can't tell	Yes	Discussion of the implications of the findings is limited and it is stated that the findings are not transferable.
Richards, 2018	Yes	Yes	Yes	Yes	Yes	Yes	No	Yes	Yes	Implications for practice are discussed but future areas of research are not outlined.
Shorey, 2019	Yes	Yes	Yes	Yes	Yes	No	Yes	Yes	Yes	The study findings are discussed against relevant literature and implications for future research and clinical practice are discussed.
Shorey, 2024	Yes	Yes	Yes	Yes	Yes	No	Yes	Yes	Yes	Transferability considered. Findings discussed in relation to relevant literature. Areas for future research highlighted. Recommendations for practice discussed.
Spiby, 2015	Yes	Yes	Yes	Yes	Yes	No	Yes	Yes	Yes	The implications of the findings are clearly discussed and areas for future research highlighted.
Taggart, 2000	Yes	Yes	Yes	Can't tell	Yes	Yes	Can't tell	Can't tell	Can't tell	The study findings can support with the designing of future volunteer interventions which suggests some transferability. Findings are discussed in relation to findings of studies of similar interventions.

Supplement 3 – ENTREQ reporting checklist

Enhancing transparency in reporting the synthesis of qualitative research: the ENTREQ statement. Table adapted form: Tong A, Flemming K, McInnes E, Oliver S, Craig J. Enhancing transparency in reporting the synthesis of qualitative research: ENTREQ. BMC Med Res Methodol. 2012;12(1):181.

No	Item	Guide and description	
1	Aim	State the research question the synthesis addresses.	Page 4 &5
2	Synthesis methodology	Identify the synthesis methodology or theoretical framework which underpins the synthesis, and describe the rationale for choice of methodology (e.g. meta-ethnography, thematic synthesis, critical interpretive synthesis, grounded theory synthesis, realist synthesis, meta-aggregation, meta-study, framework synthesis).	Page 5
3	Approach to searching	Indicate whether the search was pre-planned (comprehensive search strategies to seek all available studies) or iterative (to seek all available concepts until they theoretical saturation is achieved).	Page 5
4	Inclusion criteria	Specify the inclusion/exclusion criteria (e.g. in terms of population, language, year limits, type of publication, study type).	Page 6 & 7
5	Data sources	Describe the information sources used (e.g. electronic databases (MEDLINE, EMBASE, CINAHL, psycINFO, Econlit), grey literature databases (digital thesis, policy reports), relevant organisational websites, experts, information specialists, generic web searches (Google Scholar) hand searching, reference lists) and when the searches conducted; provide the rationale for using the data sources.	Page 5 & 6
6	Electronic Search strategy	Describe the literature search (e.g. provide electronic search strategies with population terms, clinical or health topic terms, experiential or social phenomena related terms, filters for qualitative research, and search limits).	Page 5 & supplement 1
7	Study screening methods	Describe the process of study screening and sifting (e.g. title, abstract and full text review, number of independent reviewers who screened studies).	Page 7

**8**	Study characteristics	Present the characteristics of the included studies (e.g. year of publication, country, population, number of participants, data collection, methodology, analysis, research questions).	Page 9-11 and Table 1
**9**	Study selection results	Identify the number of studies screened and provide reasons for study exclusion (e,g, for comprehensive searching, provide numbers of studies screened and reasons for exclusion indicated in a figure/flowchart; for iterative searching describe reasons for study exclusion and inclusion based on modifications t the research question and/or contribution to theory development).	Page 9 and Figure 1
**10**	Rationale for appraisal	Describe the rationale and approach used to appraise the included studies or selected findings (e.g. assessment of conduct (validity and robustness), assessment of reporting (transparency), assessment of content and utility of the findings).	Page 10
**11**	Appraisal items	State the tools, frameworks and criteria used to appraise the studies or selected findings (e.g. Existing tools: CASP, QARI, COREQ, Mays and Pope [25]; reviewer developed tools; describe the domains assessed: research team, study design, data analysis and interpretations, reporting).	Page 8 and Supplement 2
**12**	Appraisal process	Indicate whether the appraisal was conducted independently by more than one reviewer and if consensus was required.	Page 8
**13**	Appraisal results	Present results of the quality assessment and indicate which articles, if any, were weighted/excluded based on the assessment and give the rationale.	Page 11
**14**	Data extraction	Indicate which sections of the primary studies were analysed and how were the data extracted from the primary studies? (e.g. all text under the headings "results /conclusions" were extracted electronically and entered into a computer software).	Page 7
**15**	Software	State the computer software used, if any.	Page 6-8
**16**	Number of reviewers	Identify who was involved in coding and analysis.	Page 8
**17**	Coding	Describe the process for coding of data (e.g. line by line coding to search for concepts).	Page 8
**18**	Study comparison	Describe how were comparisons made within and across studies (e.g. subsequent studies were coded into pre-existing concepts, and new concepts were created when deemed necessary).	Page 8

**19**	Derivation of themes	Explain whether the process of deriving the themes or constructs was inductive or deductive	Page 8
**20**	Quotations	Provide quotations from the primary studies to illustrate themes/constructs, and identify whether the quotations were participant quotations of the author’s interpretation.	Page 12-28
**21**	Synthesis output	Present rich, compelling and useful results that go beyond a summary of the primary studies (e.g. new interpretation, models of evidence, conceptual models, analytical framework, development of a new theory or construct).	Page 28-32

Supplement 4 – PRISMA reporting checklist

Section and Topic	Item #	Checklist item	Location where item is reported
**TITLE**			
Title	1	Identify the report as a systematic review.	Page 1
**ABSTRACT**			
Abstract	2	See the PRISMA 2020 for Abstracts checklist.	Page 2
**INTRODUCTION**			
Rationale	3	Describe the rationale for the review in the context of existing knowledge.	Page 3 - 5
Objectives	4	Provide an explicit statement of the objective(s) or question(s) the review addresses.	Page 4-5
**METHODS**			
Eligibility criteria	5	Specify the inclusion and exclusion criteria for the review and how studies were grouped for the syntheses.	Page 6 & 7
Information sources	6	Specify all databases, registers, websites, organisations, reference lists and other sources searched or consulted to identify studies. Specify the date when each source was last searched or consulted.	Page 5-6
Search strategy	7	Present the full search strategies for all databases, registers and websites, including any filters and limits used.	Supplement 1
Selection process	8	Specify the methods used to decide whether a study met the inclusion criteria of the review, including how many reviewers screened each record and each report retrieved, whether they worked independently, and if applicable, details of automation tools used in the process.	Page 6
Data collection process	9	Specify the methods used to collect data from reports, including how many reviewers collected data from each report, whether they worked independently, any processes for obtaining or confirming data from study investigators, and if applicable, details of automation tools used in the process.	Page 6 & 7
Data items	10a	List and define all outcomes for which data were sought. Specify whether all results that were compatible with each outcome domain in each study were sought (e.g. for all measures, time points, analyses), and if not, the methods used to decide which results to collect.	Page 7-8
	10b	List and define all other variables for which data were sought (e.g. participant and intervention characteristics, funding sources). Describe any assumptions made about any missing or unclear information.	NA
Study risk of bias assessment	11	Specify the methods used to assess risk of bias in the included studies, including details of the tool(s) used, how many reviewers assessed each study and whether they worked independently, and if applicable, details of automation tools used in the process.	Page 8
Effect measures	12	Specify for each outcome the effect measure(s) (e.g. risk ratio, mean difference) used in the synthesis or presentation of results.	NA
Synthesis methods	13a	Describe the processes used to decide which studies were eligible for each synthesis (e.g. tabulating the study intervention characteristics and comparing against the planned groups for each synthesis (item #5)).	NA
	13b	Describe any methods required to prepare the data for presentation or synthesis, such as handling of missing summary statistics, or data conversions.	NA
	13c	Describe any methods used to tabulate or visually display results of individual studies and syntheses.	NA

Section and Topic	Item #	Checklist item	Location where item is reported
	13d	Describe any methods used to synthesize results and provide a rationale for the choice(s). If meta-analysis was performed, describe the model(s), method(s) to identify the presence and extent of statistical heterogeneity, and software package(s) used.	Page 8
	13e	Describe any methods used to explore possible causes of heterogeneity among study results (e.g. subgroup analysis, meta-regression).	NA
	13f	Describe any sensitivity analyses conducted to assess robustness of the synthesized results.	NA
Reporting bias assessment	14	Describe any methods used to assess risk of bias due to missing results in a synthesis (arising from reporting biases).	NA
Certainty assessment	15	Describe any methods used to assess certainty (or confidence) in the body of evidence for an outcome.	NA
**RESULTS**			
Study selection	16a	Describe the results of the search and selection process, from the number of records identified in the search to the number of studies included in the review, ideally using a flow diagram.	Figure 1 and Table 1
	16b	Cite studies that might appear to meet the inclusion criteria, but which were excluded, and explain why they were excluded.	Figure 1
Study characteristics	17	Cite each included study and present its characteristics.	Table 1
Risk of bias in studies	18	Present assessments of risk of bias for each included study.	Supplement 2
Results of individual studies	19	For all outcomes, present, for each study: (a) summary statistics for each group (where appropriate) and (b) an effect estimate and its precision (e.g. confidence/credible interval), ideally using structured tables or plots.	NA
Results of syntheses	20a	For each synthesis, briefly summarise the characteristics and risk of bias among contributing studies.	NA
	20b	Present results of all statistical syntheses conducted. If meta-analysis was done, present for each the summary estimate and its precision (e.g. confidence/credible interval) and measures of statistical heterogeneity. If comparing groups, describe the direction of the effect.	NA
	20c	Present results of all investigations of possible causes of heterogeneity among study results.	NA
	20d	Present results of all sensitivity analyses conducted to assess the robustness of the synthesized results.	NA
Reporting biases	21	Present assessments of risk of bias due to missing results (arising from reporting biases) for each synthesis assessed.	NA
Certainty of evidence	22	Present assessments of certainty (or confidence) in the body of evidence for each outcome assessed.	NA
**DISCUSSION**			
Discussion	23a	Provide a general interpretation of the results in the context of other evidence.	Page 28-31
	23b	Discuss any limitations of the evidence included in the review.	Page 32
	23c	Discuss any limitations of the review processes used.	Page 32
	23d	Discuss implications of the results for practice, policy, and future research.	Page 28-31

Section and Topic	Item #	Checklist item	Location where item is reported
**OTHER INFORMATION**			
Registration and protocol	24a	Provide registration information for the review, including register name and registration number, or state that the review was not registered.	Page 5
	24b	Indicate where the review protocol can be accessed, or state that a protocol was not prepared.	Page 5
	24c	Describe and explain any amendments to information provided at registration or in the protocol.	NA
Support	25	Describe sources of financial or non-financial support for the review, and the role of the funders or sponsors in the review.	Not in manuscript as per journal requirements
Competing interests	26	Declare any competing interests of review authors.	Not in manuscript as per journal requirements
Availability of data, code and other materials	27	Report which of the following are publicly available and where they can be found: template data collection forms; data extracted from included studies; data used for all analyses; analytic code; any other materials used in the review.	NA

From: Page MJ, McKenzie JE, Bossuyt PM, Boutron I, Hoffmann TC, Mulrow CD, et al. The PRISMA 2020 statement: an updated guideline for reporting systematic reviews. BMJ 2021;372:n71. doi: 10.1136/bmj.n71

For more information, visit: http://www.prisma-statement.org/
